# Supplementary material for: Dimensions of wisdom perception across twelve countries on five continents
Source: Nat Commun. 2024 Aug 14;15:6375. doi: 10.1038/s41467-024-50294-0 (PMC11324649; doi:10.1038/s41467-024-50294-0)
Supplement: Supplementary file 1 — Supplementary Information [file 41467_2024_50294_MOESM1_ESM.pdf]

Supplementary Information for
Dimensions of Wisdom Perception Across Twelve Countries on Five Continents

Rudnev, M.[1†], Barrett, H.C.[2], Buckwalter, W.[3], Machery, E.[4,5], Stich, S.[6], Barr, K.[4], Bencherifa, A.[7,5], Clancy, R.F.[8], Crone, D.L.[9], Deguchi, Y.[10], Fabiano, E.[11], Fodeman, A.D.[12], Guennoun, B.[13], Halamová, J.[14], Hashimoto, T.[15], Homan, J.[16], Kanovský, M.[14], Karasawa, K.[17], Kim, H.[18], Kiper, J.[19], Lee, M.[20], Liu, X.[21], Mitova, V.[5], Nair, R.B.[22,5], Pantovic, L.[23], Porter, B.[4], Quintanilla, P.[24,5], Reijer, J.[5], Romero, P.P.[25], Singh, P.[22], Tber, S.[7], Wilkenfeld, D.A.[4], Yi, L.[4], Grossmann, I.[1,5 †].

Table of Contents

Supplementary Methods *2*

Procedure 2

Analytical Approach 5

Supplementary Note A – Dimensionality and Measurement Invariance *7*

Preliminary analyses – item selection 7

Number of factors 9

Testing isomorphism 14

Method factor at the between-individual level 15

Unidimensionality in the ML-CFA 17

Measurement invariance tests 19

Supplementary Note B – Associations of the two dimensions of wisdom perception and the explicit ratings of wisdom *21*

Correlations between dimensions of wisdom perception and explicit ratings of wisdom 21

Predicting explicit wisdom ratings with the two dimensions of wisdom perception 23

Interactions between the two dimensions of wisdom perception in predicting explicit ratings of wisdom, knowledgeability, and understanding 27

Robustness checks of regressions of ratings of wisdom, knowledgeability and understanding on the two dimensions of wisdom perception 30

Supplementary Note C – Target rankings *37*

General ranking 37

Stability across cultural regions 39

Supplementary Note D – Measurement invariance of the wisdom probes *41*

Supplementary Methods

Procedure

Participants were presented with pairs of ten targets and then asked a series of questions about the pairs (full wording for each target is in Table S1). Each participant was assigned a reference target from the list. Participants then saw individual pairs constructed between that reference target and each of the remaining individual comparison targets. Thus, each participant saw nine pairs in total. The comparison target was selected and paired with the first target in one of two consecutive orders: ascending order or descending order, affording pseudo-randomization. For example, participant 1 would see pairs 1 vs. 2, 1 vs. 3… 1 vs. 10; participant 2 would see pairs 2 vs. 10, 2 vs. 9… 2 vs. 1; participant 3 would see 3 vs. 1, 3 vs. 2…, 3 vs.10, and so on. To facilitate the administration of the procedure, in Japan and two Indian samples (Meitei and Tamil) the number of targets was reduced to five, namely, three age groups, *religious person*, and *you*. Directly below each pairing, participants were asked the following:

> *Consider that these two characters are trying to make a difficult choice that there is no clear right or wrong answer to (for example, an important life decision). In your view, how likely is it that [REFERENCE TARGET'S NAME] will do the following things compared to [COMPARISON TARGET'S NAME]?*

Comparison characteristics were randomly listed in a matrix table below this question and judgments about them were collected using a 5-item scale labeled in the following way:

- *"[REFERENCE TARGET'S NAME] is much less likely than [COMPARISON TARGET'S NAME]",*
- *"[REFERENCE TARGET'S NAME] is moderately less likely than [COMPARISON TARGET'S NAME]",*
- *"[REFERENCE TARGET'S NAME] is equally likely than [COMPARISON TARGET'S NAME]",*
- *"[REFERENCE TARGET'S NAME] is moderately more likely than [COMPARISON TARGET'S NAME]",*
- *"[REFERENCE TARGET'S NAME] is much more likely than [COMPARISON TARGET'S NAME]".*

Table S1. List of targets

Label	Wording*
Self	When you look in the mirror, please consider how you would compare with the other character presented.
12-year-old	Payton is a twelve-year-old child who lives with their mother and father.
45-year-old	John is a forty-five-year-old man who lives with his wife and two children.
75-year-old	Pat is a seventy-five-year-old person who has a lot of life experience and many stories to share.
Religious	Elliot is a very religious person.**
Fair	Taylor is a just and fair person who puts themselves forward to fight for the rights of others.
Teacher	Alexis is a school teacher who educates twelve-year-olds about local history and literature.
Politician	Kendell is an elected political leader (e.g. chief, mayor) who represents hundreds of people.
Scientist	Dr. Morgan is a scientist who gathers information about plants, animals, and people to make sense of the world.
Doctor	Dr. Kerry is a medical doctor who has been healing people for fifteen years.

* Names are as they were used in Canadian survey. The teams across regions of data collection selected culturally appropriate names of the targets.

** Since religious practices and attributes widely vary across cultures, the description of the religious person was short to increase cross-cultural comparability.
Researchers from each site picked the gender of the targets deemed culturally appropriate and the wording of items was changed accordingly (e.g., *doctor* was female in Morocco, Slovakia, and India, male in China, and gender-neutral in the other samples (Table S2).

Table S2. Gender of targets by sample

	12-y.o.	45-y.o.	75-y.o.	Religious	Teacher	Fair	Politician	Scientist	Doctor
Canada (English)	N	M	N	N	N	N	N	N	N
USA	N	M	N	N	N	N	N	N	N
Ecuador (Spanish)	M	F	M	M	F	M	N	N	N
Peru (Spanish)	M	F	M	M	F	M	N	N	N
Morocco	F	M	M	M	F	M	N	M	F
India (Hindi)	N	F	M	M	F	M	F	N	F
India (Meitei)	N	F	M	M	-	-	-	-	-
India (Tamil)	N	F	M	M	-	-	-	-	-
China	N	M	M	N	N	M	M	M	M
Korea	F	M	N	M	N	F	M	N	N
Japan	N	M	N	N	-	-	-	-	-
South Africa (Afrikaans)	F	M	M	M	N	F	M	N	N
South Africa (isiZulu)	F	M	M	F	N	F	M	N	N
South Africa (Sepedi)	F	M	M	M	N	M	N	N	N
Slovakia	F	M	M	F	M	M	F	F	F

Note. F – female, M – male, N – gender-neutral term, dash means the target was not used in the sample.

We chose the specific age of 12 to represent a target who is sufficiently cognitively developed to understand high-level constructs but below the threshold for a stereotypical teenager. We chose a *75-year-old* as a representative of an older person across societies (past the common threshold of retirement age of 65 in thematic World Bank/UN data sources). We selected a *teacher*, a *scientist*, and a *doctor*, because they are explicitly mentioned as exemplars of wisdom in prior North American literature [e.g., 1]. Finally, we selected a *politician* and a *fair person* (who is an activist fighting for human rights) as exemplars of civic leadership—another domain often associated with wisdom [1]. All characteristics are listed in Table S3. In the samples with the reduced number of targets, we picked *religious* person and *75-year-old* as exemplars of wisdom using prior empirical research, *self* is neutral in this regard and *12-* and *45-year-olds* are non-wise exemplars, so the set of targets could be considered balanced.

Table S3. List of characteristics used to compare targets.

Label	Full item wording
think before acting	think before acting or speaking
think in many ways	think about the issue in many different ways
think logically	think logically (i.e., provide rational, systematic argument to support their choices)
apply experiences	apply what they have learned from life experiences
control of emotions	have good control of emotions
hide emotions	not show emotions
recognize change	recognize that events are in flux and can change
benefit for their group	maximize the benefit for their group, regardless of the cost for others
care for others' feelings	care for others' feelings
(intellectual) humility	show humility (i.e., think that they could be wrong)
pay attention to emotions	pay attention to what their emotions are telling them
others' perspective	consider someone else's perspective
neutral advice	rely on neutral third parties for advice
sense of humor	respond with a sense of humor
pay attention to divinity	pay attention to what nature or divinity is telling them
aware of bodily expressions	be aware of others' facial and bodily expressions
[excluded]	disengage from the situation and let it unfold as it does
[excluded]	show pride in themselves
[excluded]	notice if their body tenses up or relaxes when thinking about different options

Note: excluded – characteristics omitted from analyses for theoretical or modeling reasons.

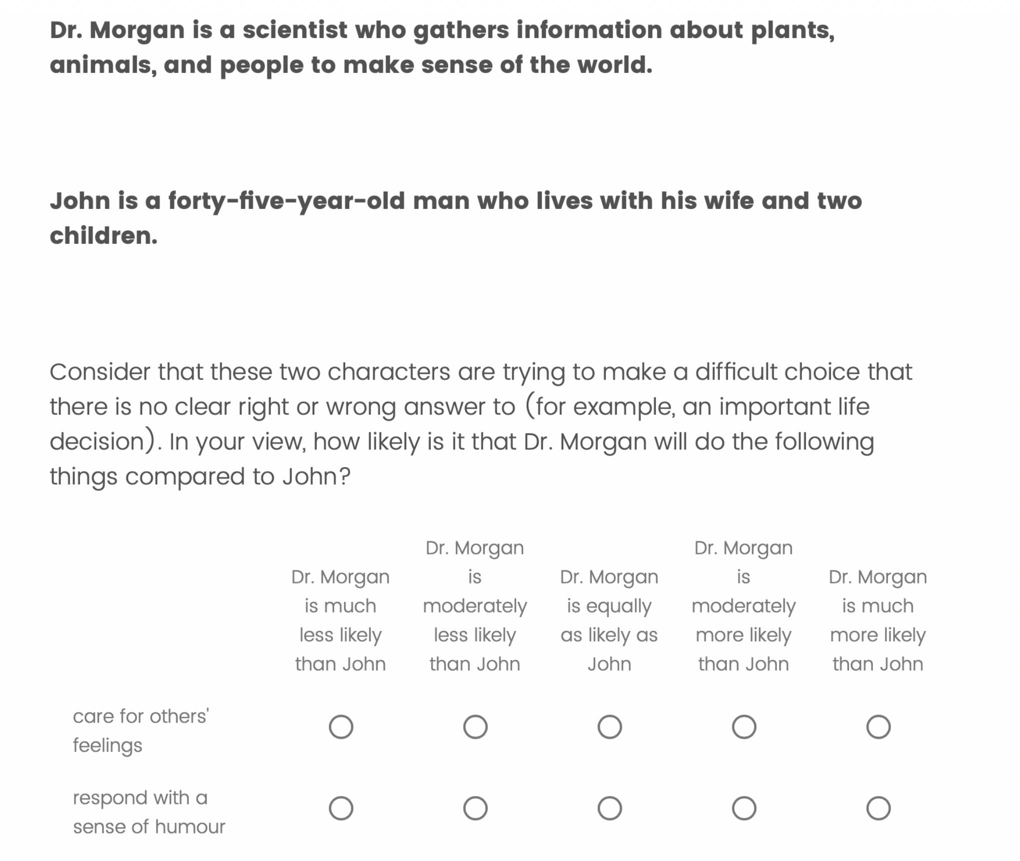

Dr. Morgan is a scientist who gathers information about plants, animals, and people to make sense of the world.

John is a forty-five-year-old man who lives with his wife and two children.

Consider that these two characters are trying to make a difficult choice that there is no clear right or wrong answer to (for example, an important life decision). In your view, how likely is it that Dr. Morgan will do the following things compared to John?

	Dr. Morgan is much less likely than John	Dr. Morgan is moderately less likely than John	Dr. Morgan is equally as likely as John	Dr. Morgan is moderately more likely than John	Dr. Morgan is much more likely than John
care for others' feelings	○	○	○	○	○
respond with a sense of humour	○	○	○	○	○

Figure S1. An example of the probes in the Qualtrics questionnaire fielded in Canada.

Table S4. Parental education and religiosity of participants across samples.

	Parental education, %			Importance of religion		
	Primary + secondary	Some university	University degree	"not at all" + "a little bit" (%)	"very" + "quite" (%)	Mean (1-5)
China	.28	.28	.34			
India	.57	.02	.37	.26	.42	3.25
Korea & Japan	.52	.15	.29	.81	.07	1.70
Morocco	.38	.06	.26	.07	.80	4.22
North America	.06	.13	.43	.61	.22	2.24
Slovakia	.51	.45	.04	.47	.40	2.95
South Africa	.22	.32	.43	.11	.77	4.21
South America	.19	.18	.60	.46	.32	2.76

Analytical Approach

Each model we explored included a two-level structure (see Fig. 6 in the main text), though specifics varied. At the first stage we employed a multilevel exploratory factor analysis (ML-EFA). After identifying the optimal factor solution, we built a multilevel confirmatory factor analysis model (ML-CFA). Next, we extended this model to multilevel structural equation models (ML-SEM), to estimate associations between identified latent factors (Reflective Orientation and Socio-Emotional Awareness) and explicit ratings of wisdom, knowledgeability, and understanding. Finally, we extended the ML-CFA model to a multiple groups model, simultaneously estimating the ML-CFA model fit in eight subsamples. This procedure allowed us to test whether there were differences between cultural regions by estimating equivalence of factor loadings, and by comparing associations between factors and wisdom, knowledgeability, and understanding ratings as well as targets' positions along the latent dimensions of wisdom perception.

In addition to CFI and RMSEA, to compare models we examined differences in information criteria (sample-adjusted Bayesian information criterion – BIC). We did not consider *p*-values of the model χ^2 because they are not informative in very large samples. We used Bayesian Gibbs' sampler random walk algorithm to estimate models that included an interaction between the latent variables. The following criteria indicated the appropriate quality of the Bayesian models: potential scale reduction factor < 1.1; effective sample size > 300 for each parameter; in addition, we visually examined the autocorrelation plots and the traceplots for each parameter to ensure convergence between chains [2,3].

Beside Mplus software, we used R environment[4] and the following packages: *cowplot*[5], *dplyr*[6], *ggraph*[7], *ggrepel*[8], *ggtext*[9], *glmnet*[10], *gridExtra*[11], *hdlm*[12], *kableExtra*[13], *knitr*[14], *LittleHelpers*[15], *MplusAutomation*[16], *psych*[17], *qgraph*[18], *reshape2*[19], *showtext*[20], *tidygraph*[21], *tidyr*[22], *tidyselect*[23].

Table S5. Sample of the data for the multilevel analysis.

id	Reference	Comparison	Targets' characteristics: apply experiences	think in many ways	...	hide emotions	Reference Wisdom	Comparison Wisdom	*D*
1	$45_{\text{year old}}$	$12_{\text{year old}}$	4	4		4	3	2	1
1	$45_{\text{year old}}$	$45_{\text{year old}}$	3	3		3	3	3	0
1	$45_{\text{year old}}$	$75_{\text{year old}}$	3	2		2	3	4	-1
1	$45_{\text{year old}}$	Doctor	3	2		2	3	4	-1
1	$45_{\text{year old}}$	Fair	3	2		3	3	5	-2
1	$45_{\text{year old}}$	Politician	2	1		2	3	4	-1
1	$45_{\text{year old}}$	Religious	3	4		2	3	2	1
1	$45_{\text{year old}}$	Scientist	3	2		2	3	3	0
1	$45_{\text{year old}}$	Teacher	3	2		2	3	3	0
1	$45_{\text{year old}}$	you	3	2		2	3	3	0
10	Teacher	$12_{\text{year old}}$	3	3		3	5	3	0
10	Teacher	$45_{\text{year old}}$	3	3		3	5	3	0

Note: *id* = participant's id. *D* = difference between wisdom attributed to the reference and comparison targets.

Supplementary Note A – Dimensionality and Measurement Invariance

Preliminary analyses – item selection

We ran a series of ML-EFA models with 1-5 factors at each level. We did not consider a higher number of factors. We considered oblique *Geomin* rotated loadings because all the items were meant to measure similar construct(s).

Items "consider someone else's perspective" and "be aware of others' facial and bodily expressions" showed variable loadings across cultural regions. Another item ("show pride in themselves") was the only reverse-coded characteristic (opposite of humility), and thus inconsistent with the others. The variability of loadings was reduced after removing these items. Although variability persisted in regard to another three items (formal tests of measurement invariance across regions rejected equality of loadings, see below), we decided to keep these items in the analysis for a greater content coverage.

Table S6. Factor loadings from the five-factor ML-EFA fitted with 19 items, within-individual part.

<table>
<tr><th></th><th>F1</th><th>F2</th><th>F3</th><th>F4</th><th>F5</th></tr>
<tr><td>think logically (i.e., provide rational, systematic argument to support their choices)</td><td>.67</td><td>-.02</td><td>-.05</td><td>-.03</td><td>.14</td></tr>
<tr><td>think before acting or speaking</td><td>.66</td><td>.04</td><td>.04</td><td>-.01</td><td>.01</td></tr>
<tr><td>have good control of emotions</td><td>.63</td><td>.01</td><td>.05</td><td>.06</td><td>-.09</td></tr>
<tr><td>think about the issue in many different ways</td><td>.58</td><td>.01</td><td>.04</td><td>-.04</td><td>.17</td></tr>
<tr><td>recognize that events are in flux and can change</td><td>.48</td><td>.03</td><td>.11</td><td>.08</td><td>.04</td></tr>
<tr><td>apply what they have learned from life experiences</td><td>.45</td><td>.01</td><td>.24</td><td><|.01|</td><td>-.08</td></tr>
<tr><td>not show emotions</td><td>.44</td><td>-.21</td><td>-.05</td><td>.18</td><td>-.05</td></tr>
<tr><td>consider someone else's perspective</td><td>.23</td><td>.24</td><td>.15</td><td>.06</td><td>.25</td></tr>
<tr><td>show humility (i.e., think that they could be wrong)</td><td>.18</td><td>.39</td><td>.04</td><td>.20</td><td>.06</td></tr>
<tr><td>care for others' feelings</td><td>.04</td><td>.36</td><td>.36</td><td>.01</td><td>.03</td></tr>
<tr><td>be aware of others' facial and bodily expressions</td><td>.19</td><td>.02</td><td>.52</td><td>-.14</td><td>.02</td></tr>
<tr><td>notice if their body tenses up or relaxes when thinking about different options</td><td>.16</td><td>-.08</td><td>.47</td><td>-.03</td><td>.02</td></tr>
<tr><td>maximize the benefit for their group, regardless of the cost for others</td><td>.04</td><td>-.34</td><td>.35</td><td>.06</td><td>.06</td></tr>
<tr><td>pay attention to what nature or divinity is telling them</td><td>.03</td><td>.09</td><td>.31</td><td>.18</td><td>-.27</td></tr>
<tr><td>show pride in themselves</td><td>.02</td><td>-.42</td><td>.37</td><td>.01</td><td>.05</td></tr>
<tr><td>pay attention to what their emotions are telling them</td><td>-.16</td><td>.06</td><td>.58</td><td>.08</td><td>-.04</td></tr>
<tr><td>disengage from the situation and let it unfold as it does</td><td>.01</td><td>-.06</td><td>.02</td><td>.48</td><td><|.01|</td></tr>
<tr><td>rely on neutral third parties for advice</td><td>.03</td><td>.05</td><td>.03</td><td>.25</td><td>.35</td></tr>
<tr><td>respond with a sense of humour</td><td>-.03</td><td>.04</td><td>.19</td><td>.22</td><td>.09</td></tr>
</table>

Note. Loadings higher than .3 in magnitude are in bold font.

Table S7. Factor loadings from a two-factor ML EFA fitted with 19 items within each cultural region, within-individual part.

	Factor	CN	IND	KJ	MO	NA	SAF	SAM	SK
apply what they have learned from life experiences	1	.24	**.65**	.26	.10	**.52**	**.54**	**.57**	**.71**
	2	**.37**	-.03	**.38**	**.55**	.15	.04	.09	.09
be aware of others' facial and bodily expressions	1	.17	**.60**	**.31**	.13	**.39**	**.54**	**.46**	-.01
	2	**.51**	.04	.20	**.55**	**.36**	.01	.25	**.87**
care for others' feelings	1	-.10	**.56**	**.70**	**.64**	.01	**.49**	<|.01|	**.38**
	2	**.79**	-.02	.02	.04	**.68**	.01	**.65**	**.46**
consider someone else's perspective	1	.01	**.60**	**.51**	.27	**.40**	**.55**	**.33**	**.32**
	2	**.74**	<|.01|	.30	.04	**.42**	-.01	**.32**	**.49**
disengage from the situation and let it unfold as it does	1	**.59**	.05	**.31**	.09	.10	.04	-.01	.11
	2	.07	**.37**	-.16	**-.38**	.10	**.41**	.07	.22
have good control of emotions	1	**.47**	**.63**	.19	-.09	**.70**	**.58**	**.60**	**.77**
	2	.30	.01	**.51**	**.60**	-.03	-.02	.06	.01
maximize the benefit for their group, regardless of the cost for others	1	.23	-.02	-.22	-.15	.24	.23	**.34**	**.40**
	2	-.10	**.50**	.18	**.33**	-.02	.21	-.06	-.01
not show emotions	1	**.45**	**.33**	<|.01|	-.27	**.47**	.12	**.46**	**.60**
	2	.24	.18	**.52**	**.40**	**-.40**	.25	**-.36**	-.05
notice if their body tenses up or relaxes when thinking about different options	1	**.30**	**.41**	**.35**	.15	**.40**	**.43**	**.49**	.10
	2	.25	.15	.15	.28	.22	.10	.17	**.79**
pay attention to what nature or divinity is telling them	1	.23	**.49**	.28	**.39**	-.01	**.40**	-.08	.15
	2	.04	-.01	-.16	.01	.28	.06	**.38**	**.37**
pay attention to what their emotions are telling them	1	.28	**.48**	**.45**	**.46**	-.05	**.37**	.02	-.07
	2	.28	.11	-.09	-.19	**.55**	.12	**.55**	**.70**
recognize that events are in flux and can change	1	**.72**	**.61**	**.39**	.07	**.60**	**.48**	**.54**	**.57**
	2	<|.01|	.04	**.36**	**.56**	.13	.03	.10	.23
rely on neutral third parties for advice	1	**.35**	.14	**.32**	.20	**.31**	.13	.15	.09
	2	.19	.28	.14	-.08	.05	**.31**	.29	**.39**
respond with a sense of humour	1	.26	.27	**.35**	**.43**	.01	-.07	-.02	**.35**
	2	.27	.11	-.07	-.16	**.34**	**.48**	.28	.24
show humility (i.e., think that they could be wrong)	1	**.32**	**.51**	**.61**	**.57**	.13	**.41**	.01	**.32**
	2	**.34**	-.04	-.01	.06	**.50**	.08	**.53**	**.43**
show pride in themselves	1	-.11	.01	-.13	**-.33**	.26	.11	**.41**	**.45**
	2	-.19	**.44**	.30	.24	-.20	.30	-.23	<|.01|
think about the issue in many different ways	1	**.77**	**.64**	.21	-.01	**.67**	**.53**	**.64**	**.80**
	2	-.11	<|.01|	**.52**	**.68**	.07	<|.01|	-.02	-.01
think before acting or speaking	1	**.66**	**.69**	.28	<|.01|	**.71**	**.64**	**.69**	**.68**
	2	.11	-.04	**.50**	**.71**	.01	-.09	.01	.13
think logically (i.e., provide rational, systematic argument to support their choices)	1	**.76**	**.62**	-.02	.10	**.80**	**.58**	**.75**	**.85**
	2	-.18	<|.01|	**.71**	**.63**	-.14	-.06	-.17	-.09

Note. Loadings higher than .3 in magnitude are in bold font. CN – China, IND – India, KJ – Korea & Japan, MO – Morocco, NA – North America, SAF – South Africa, SAM – South America, SK – Slovakia.

Number of factors

ML-EFA suggested several well-fitting multi-factor solutions; a few of them were parsimonious and revealed factors that were easily interpretable based on their loadings.

Five-factor solution. Following model fit indices only (see Table S8), the best solution suggested by the ML-EFA involved five factors. When considering the best-fitting factor solution with 5 factors at both levels, the fifth factor had only two substantive (>|.2|) loadings on items "rely on neutral third parties for advice" and "maximize the benefit for their group." The fourth factor had two non-negligible factor loadings as well ("pay attention to emotions" and "pay attention to divinity"), see Table S6 for all factor loadings. It appeared that any commonality between the latter two items beyond (the one captured by the first two factors) was their specific wording containing "paying attention." The third factor in this solution also had only few non-negligible loadings, the largest of which were for items "have good control of emotions" and "not show emotions." Such factors in EFA do not capture substantively meaningful variance in participants' ratings beyond the method of measurement (e.g., similarity in wordings). In a CFA framework, such factors could be effectively replaced with residual covariances making the solution more parsimonious [24]. In contrast, the first and the second factor had multiple large loadings which formed a meaningful pattern (see below). Therefore, the fourth and fifth factors were excessive. On these grounds, we rejected the 5-factor solution.

Four-factor solution. The fourth factor in the four-factor solution revealed only two non-negligible factor loadings: "not show emotions" and "maximize the benefit for their group." In the same vein, the third factor loaded on "rely on neutral third parties" and "consider someone else's perspective." The first and the second factor had multiple large loadings which formed a meaningful pattern. We considered two non-negligible loadings per factor to be a sign of a residual correlation rather than a substantive latent variable, especially so when the combination of the items did not make much substantive sense. Therefore, the third and fourth factors were superfluous, and we also rejected the four-factor solution.

Three-factor solution. The third factor in the three-factor solution had four non-negligible factor loadings: "think about the issue in many different ways," "think logically (i.e., provide rational, systematic argument to support their choices)," "consider someone else's perspective," and a negative loading on "pay attention to what nature or divinity is telling them." This factor could be considered to be meaningful. The first two factors were similar to the solution in the two-factor solution.

Two-factor solution. Both factors in this solution had multiple and large factor loadings. See the main text for the interpretation of the two factors. Beside the one-factor solutions, the two-factor solutions were the most parsimonious yet well-fitting.

One-factor solution. ML-EFA based on 16 items revealed a poor fit of a unidimensional model at both levels. One-factor models at either within- or between-individual level had sufficient fit, as did all the other models, but in general one-factor models showed inferior fit statistics. Moreover, the difference in fit indices between 1- and 2-factor models at the within-individual level was relatively large: ΔCFI > .3, Δ RMSEA > .1 pointing to the inferiority of the unidimensional model. We revisit the issue again with the confirmatory models below.

Table S8. Fit indices for the pooled sample ML-EFA models based on 16 items (solutions for 1 to 5 factors at each level).

*N*f Between	*N*f Within	χ^2	*df*	CFI	BIC	RMSEA
5	5	527.3	100	.993	1089071	.013
4	5	703.5	112	.991	1089223	.014
3	5	893.8	125	.988	1089416	.016
5	4	943.3	112	.987	1089541	.017
2	5	1083.6	139	.985	1089648	.016
4	4	1206.0	124	.983	1089720	.019
3	4	1874.7	137	.973	1089969	.022
5	3	1275.2	125	.982	1089977	.019
4	3	1449.8	137	.980	1090157	.019
2	4	1509.0	151	.979	1090200	.019
3	3	1721.7	150	.976	1090447	.020
2	3	1963.5	164	.972	1090773	.021
5	2	1852.2	139	.973	1090860	.022
4	2	2015.9	151	.971	1091070	.022
1	5	2189.5	154	.968	1091237	.023
3	2	2502.1	164	.964	1091437	.024
1	4	2588.4	166	.962	1091776	.024
2	***2***	***2639.8***	***178***	***.962***	***1091880***	***.023***
1	3	3026.8	179	.956	1092337	.025
1	2	3614.2	193	.947	1093417	.026
5	1	5656.9	154	.914	1096756	.038
4	1	5807.3	166	.912	1096971	.037
3	1	6461.1	179	.902	1097345	.037
2	1	6330.3	193	.904	1097790	.035
1	1	8412.0	208	.872	1101168	.039

Note. *N*f = Number of factors. Selected model is bolded and in italics. All χ^2 p < .001.

To choose one of the solutions, we examined the congruence of factor loadings across different solutions. Such congruence analyses indicated that the first two factors (within-individual level) were similar across all two-, three-, four-, and five-factor solutions (see Table S9): the first factor's loadings from a two-factor solution was congruent with factor loadings in one-, three-, four- and five-factor solutions r_c = .88, .97, .99, and .87; loadings of the second factor from the two-factor solution showed very high degree of overlap with the corresponding factor loadings from three-, four- and five-factor solutions, r_c = .99, .97, and .87. However, loadings beyond the two factors revealed substantial incongruence. Therefore, the best-fitting five- and four-factor models could be efficiently represented in the CFA fashion as a two-factor model with one or two residual covariances. This is especially effective as the two factors were easy to interpret.

The third factor from the three-factor model (see Table S9) could be a candidate for the third dimension of wisdom perception. However, this factor conceptually overlapped with the first factor (Reflective Orientation) and in a CFA framework it would represent a subset of this factor with multiple cross-loadings.

Table S9. Factor loadings from the three-factor pooled-sample ML-EFA fitted with 16 items, within-individual part.

	F1	F2	F3
care for others' feelings	**.68**	-.03	-.05
show humility (i.e., think that they could be wrong)	**.56**	.01	.02
pay attention to what their emotions are telling them	**.52**	<|.01|	-.13
consider someone else's perspective	**.49**	.09	.21
respond with a sense of humour	**.33**	.01	-.01
pay attention to what nature or divinity is telling them	**.32**	.13	-.25
be aware of others' facial and bodily expressions	**.31**	**.33**	.01
rely on neutral third parties for advice	**.31**	-.01	.19
apply what they have learned from life experiences	.14	**.51**	-.01
recognize that events are in flux and can change	.14	**.47**	.13
think about the issue in many different ways	.05	**.51**	**.32**
think before acting or speaking	.02	**.65**	.10
have good control of emotions	<|.01|	**.68**	-.03
maximize the benefit for their group, regardless of the cost for others	-.04	**.29**	<|.01|
think logically (i.e., provide rational, systematic argument to support their choices)	-.07	**.60**	**.30**
not show emotions	-.22	**.56**	-.02

Note. Loadings larger than absolute .3 are in bold.

Factor structure at the between-individual level replicated the results in regard to the first two factors but revealed a very different third factor (that is, non-isomorphic). Third factor at the between-individual level seemed to capture common variance of the items "not show emotions," "have good control of emotions," and "maximize the benefit for their group." It is possible that this factor has substantive meaning (e.g., hiding emotions in favor of group), but it seemed to be a subset of a more easily interpretable factor 1 (Reflective Orientation). Interestingly, the factor in the unidimensional factor solution was congruent with the first factor in the two-factor solution, but less so across the other solutions, suggesting that the two factors were positively correlated. All in all, the consideration of different factor solutions gave us good reasons to choose the two-factor solution at both levels.

Taking into consideration parsimony, stability across solutions with different numbers of factors, as well as interpretability of each factor, we decided to select a two-factor model, which we appended with several residual covariances, so that it effectively represents in a CFA framework the best fitting four- and five-factor EFA models.

In addition, we tested whether exclusion of samples with a shortened list of targets affected the results. This new analysis replicated the results based on full sample very closely (Table S11).

Table S10. Congruence coefficients across ML-EFA factor solutions with varying number of factors, within-individual level part.

	2-factor model, Factor 1	2-factor model, Factor 2
1-factor model, Factor 1	**.88**	.57
3-factor model, Factor 1	.17	**.99**
3-factor model, Factor 2	**.97**	.07
3-factor model, Factor 3	.59	-.17
4-factor model, Factor 1	**.99**	.12
4-factor model, Factor 2	.21	**.97**
4-factor model, Factor 3	.16	.43
4-factor model, Factor 4	.40	-.11
5-factor model, Factor 1	**.87**	.14
5-factor model, Factor 2	.18	**.87**
5-factor model, Factor 3	**.65**	-.02
5-factor model, Factor 4	.22	**.69**
5-factor model, Factor 5	.22	-.17

Note. Congruence coefficients of the first and second factors from different solutions are in bold.

Table S11 Fit indices for the pooled sample ML-EFA excluding Japanese, Meitei, and Tamil samples with a shortened list of targets (solutions for 1 to 5 factors at each level).

N_f Between	N_f Within	χ^2	*df*	CFI	BIC	RMSEA	SRMR within	SRMR between
1	1	8267.4	208	0.865	1032034	0.040	0.048	0.070
2	1	3475.8	193	0.945	1024284	0.027	0.024	0.076
3	1	2940.6	179	0.954	1023291	0.026	0.020	0.078
4	1	2584.3	166	0.959	1022774	0.025	0.017	0.079
5	1	2162.6	154	0.966	1022264	0.023	0.015	0.080
1	2	6199.4	193	0.899	1028685	0.036	0.046	0.030
**2**	**2**	**2492.9**	**178**	**0.961**	**1022742**	**0.023**	**0.021**	**0.027**
3	2	1873.9	164	0.971	1021722	0.021	0.017	0.028
4	2	1484.3	151	0.978	1021194	0.019	0.013	0.028
5	2	1052.3	139	0.985	1020669	0.017	0.010	0.029
1	3	6444.3	179	0.895	1028283	0.038	0.046	0.026
2	3	2412.8	164	0.962	1022341	0.024	0.021	0.023
3	3	1782.3	150	0.973	1021419	0.021	0.017	0.020
4	3	1471.0	137	0.978	1020920	0.020	0.014	0.021
5	3	893.8	125	0.987	1020456	0.016	0.009	0.021
1	4	6016.0	166	0.902	1027933	0.039	0.046	0.019
2	4	2008.6	151	0.969	1022000	0.023	0.021	0.016
3	4	1387.7	137	0.979	1021148	0.020	0.016	0.017
4	4	1191.6	124	0.982	1020741	0.019	0.013	0.016
5	4	711.0	112	0.990	1020275	0.015	0.009	0.015
1	5	5636.5	154	0.908	1027736	0.039	0.046	0.016
2	5	1789.0	139	0.972	1021806	0.022	0.021	0.012
3	5	1221.1	125	0.982	1020982	0.019	0.016	0.013
4	5	945.0	112	0.986	1020579	0.018	0.013	0.013
5	5	528.3	100	0.993	1020132	0.013	0.009	0.011

Note. N_f = Number of factors. Selected model is bolded and in italics. All χ^2 $p < .001$.

Testing isomorphism

The results show that the fit of isomorphic and non-isomorphic models was very similar in the pooled sample (Table S12) and each of the cultural regions (Table S13). In six regions model fit indices such as BIC and RMSEA suggested identical if not superior fit of the isomorphic models. Overall, the differences between the two models were negligible, so to arrive at the more comparable model, we opted for a more parsimonious isomorphic model.

Table S12. Statistical fit of isomorphic and non-isomorphic ML-CFA models with a method factor at the between-level estimated at the pooled sample.

Model	BIC	χ^2	*df*	CFI	TLI	RMSEA	SRMR Within	SRMR Between
Models without a method factor at the between-level								
Non-isomorphic	1,091,562	2545.7	208	.963	.958	.021	.024	.036
Isomorphic	1,092,120	3029.5	224	.956	.953	.022	.028	.082
Models with a method factor at the between-level								
Non-isomorphic	1,091,373	2517.1	207	.964	.958	.021	.024	.042
Isomorphic	1,091,354	2546.9	223	.964	.961	.020	.025	.047

Table S13. Comparison of isomorphic and non-isomorphic models (without method factor) by cultural region.

		BIC	CFI	TLI	RMSEA	$SRMR_{within}$	$SRMR_{Between}$
China	Isomorphic	87076.7	.899	.892	0.042	.047	.116
	Non-isomorphic	87073.6	.904	.890	0.042	.046	.094
India	Isomorphic	109386.2	.973	.971	0.021	.025	.069
	Non-isomorphic	109429.3	.974	.970	0.021	.023	.052
Japan & Korea	Isomorphic	97996.6	.911	.905	0.032	.041	.099
	Non-isomorphic	98024.8	.914	.900	0.033	.039	.071
Morocco	Isomorphic	73017.9	.926	.921	0.029	.050	.124
	Non-isomorphic	73070.0	.897	.883	0.035	.049	.122
North America	Isomorphic	194187.7	.908	.901	0.034	.052	.116
	Non-isomorphic	194201.3	.910	.897	0.035	.051	.100
South Africa	Isomorphic	210396.6	.968	.966	0.018	.022	.079
	Non-isomorphic	210407.9	.972	.968	0.017	.020	.049
South America	Isomorphic	142531.8	.895	.887	0.033	.049	.127
	Non-isomorphic	142582.6	.894	.877	0.035	.048	.128
Slovakia	Isomorphic	89931.5	.903	.896	0.042	.044	.136
	Non-isomorphic	89862.3	.907	.894	0.042	.048	.183

Table S14. Standardized factor loadings (unstandardized loadings are fixed to be equal at the between and within levels) estimated by the ML CFA at the pooled sample.

	Reflective Between	Reflective Within	Socio-Emotional Awareness Between	Socio-Emotional Awareness Within
think before acting[a]	**.97**	**.67**		
think in many ways[b]	**.96**	**.65**		
recognize change	**.95**	**.64**		
control of emotions[a,c]	**.92**	**.61**		
think logically[b]	**.91**	**.64**		
apply experiences	**.86**	**.61**		
hide emotions[c]	**.65**	**.38**		
benefit for their group	**.47**	.29		
aware of bodily expressions	**.52**	**.36**	**.43**	.27
care for others' feelings			**.95**	**.62**
(intellectual) humility			**.92**	**.57**
pay attention to emotions[d]			**.88**	**.50**
neutral advice[e]			**.69**	**.37**
others' perspective[e]	**.33**	.22	**.66**	**.41**
sense of humor			**.65**	**.38**
pay attention to divinity[d]			**.57**	**.36**

Note. [a-e] Residual covariances allowed between items with the same letter superscripts.
CFI = .966, TLI = .961; RMSEA = .019; $SRMR_{within}$ = .025; $SRMR_{between}$ = .029. Loadings larger than absolute .3 are in bold.

Method factor at the between-individual level

Prior cross-cultural research suggests that differences in response styles may affect survey responses in questionnaire format similar to the one used in our instrument [25]. Given the structure of our multilevel model, individuals' response style tendencies could have effect at the between-individual level of analysis, while our chief analyses concerned the within-individual level. In other words, by design the method bias would have *only* affected the between-individual level of analysis.

Nevertheless, we sought to explore if adding a common *tau*-equivalent factor (i.e., with all its loadings constrained to be equal) representing response tendency and uncorrelated with the two substantive factors at the between-individual level improved the model fit. To this end, we extended the isomorphic model at the between-individual level to a bifactor model with the loadings of the common factor fixed to 1. Table S12 shows that the introduction of the method factor to the isomorphic model only resulted in a modest improvement of the model fit for the pooled sample. The improvement was particularly noticeable in information criteria (BIC) and $SRMR_{between}$. The limited improvement was expected because up to 80% of variance in the data manifested at the within-individual level, while the common method factor addition solely concerned the between-individual level of analysis. In contrast, comparison of $SRMR_{between}$ for model with (.042) and without the method factor (.082) resulted in an improved model fit.

Comparison of models with and without the method factor in each cultural region (Table S15) showed that the former improved model in every cultural region. However, the method

factor loadings were small and mostly insignificant (Table S16) with a possible exception of South Africa. Given that the method factor was not necessary for an unbiased estimate of responses at the within-individual level of analysis, we chose a more parsimonious model without it.

Table S15. Comparing isomorphic models with method factor at the between-individual level and without it, by cultural region.

		BIC	CFI	TLI	RMSEA	$SRMR_{within}$	$SRMR_{Between}$
China	No method	87076.7	.899	.892	.042	.047	.116
	with method	86954.6	.913	.900	.040	.046	.086
India	No method	109386.1	.973	.971	.021	.025	.069
	with method	109393.2	.977	.974	.020	.025	.054
Japan & Korea	No method	97996.6	.911	.905	.032	.041	.099
	with method	97897.7	.924	.913	.030	.041	.088
Morocco	No method	73017.9	.926	.921	.029	.050	.124
	with method	72998.8	.936	.927	.027	.050	.071
North America	No method	194187.7	.908	.901	.034	.052	.116
	with method	193938.3	.921	.909	.033	.050	.085
South Africa	No method	210396.6	.968	.966	.018	.022	.079
	with method	210201.5	.983	.981	.013	.021	.034
South America	No method	142531.8	.895	.887	.033	.049	.127
	with method	142406.3	.907	.893	.033	.047	.075
Slovakia	No method	89931.5	.903	.896	.042	.044	.136
	with method	89818.7	.911	.898	.041	.045	.091

Table S16. Standardized factor loadings of the common factor from group-specific two-factor bifactor models with isomorphic group factor loadings, where the group factors are allowed to correlate.

	China	India	Japan & Korea	Morocco	North America	South Africa	South America	Slovakia
think logically	**.70**	.11	**-.41**	.17	**.50**	**.39**	**-.63**	.15
think in many ways	**.56**	.14	.15	.18	.26	**.47**	**-.41**	.16
think before acting	**.55**	.11	-.19	.23	**.38**	.27	**-.42**	.17
hide emotions	**.51**	**.47**	**-.59**	**.51**	**.85**	**.75**	**-.74**	.15
benefit for their group	**.48**	**.95**	-.29	-.01	.14	**.70**	**-.31**	**.36**
recognize change	**.45**	.06	.12	.20	.25	**.49**	-.16	**.48**
control of emotions	**.41**	.11	**-.55**	**.31**	**.34**	.28	**-.41**	.17
neutral advice	.23	.29	.07	**-.68**	.25	**.64**	.11	**.74**
pay attention to divinity	.16	.18	-.11	.03	-.12	.22	**.58**	.22
pay attention to emotions	.12	.20	.28	**-.50**	**-.47**	.29	**.66**	**.78**
aware of bodily expressions	.07	.15	.13	.20	-.15	.26	-.06	**.48**
(intellectual) humility	.01	-.04	-.02	-.17	-.16	.18	**.43**	.29
sense of humor	-.06	.21	.27	**-.86**	**-.34**	**.48**	**.40**	**.65**
apply experiences	-.15	.08	.08	**.32**	.14	.28	-.13	.12
others' perspective	-.23	.12	.07	**-.54**	-.02	.26	.18	**.41**
care for others' feelings	**-.32**	.09	.28	-.02	**-.48**	.11	**.67**	.20

Note. Loadings larger than absolute .3 are in bold.

Unidimensionality in the ML-CFA

Though our exploratory analyses suggested that a single factor solution had lower fit to the data, the correlations between the latent factors from a two-factor solution were moderate to high and positive, r (pooled sample) = .68, $.36 \leq r$(cultural region) < .88 (see Table S17). Therefore, we decided to explicitly compare a single- and two-factor solutions. However, the results in Table S18 show that in all countries the two-factor solution fit the data better than the one-factor solution. Therefore, we continued to proceed with the two-factor model.

Table S17. Correlations between the two factors based on the multiple group (partial metric invariance multigroup multilevel confirmatory factor analysis model with isomorphic loadings) model and ML-CFA model at the pooled sample.

	Between	Within
China	.83 [.70 .95]	.84 [.79 .88]
India	.85 [.79 .92]	.88 [.84 .92]
Japan & Korea	.85 [.74 .95]	.75 [.69 .81]
Morocco	.26 [.02 .49]	.33 [.24 .43]
North America	.61 [.50 .73]	.58 [.52 .63]
South Africa	.91 [.86 .96]	.83 [.78 .87]
South America	.36 [.18 .53]	.41 [.32 .49]
Slovakia	.65 [.50 .80]	.77 [.73 .81]
Pooled sample	.82 [.79 .85]	.69 [.66 .71]

Note. 95%CI is reported in brackets.

Table S18. Fit indices of the one- and two-factor ML-CFA models (isomorphic, no method factor).

	N factors	BIC	CFI	TLI	RMSEA	SRMR Within	SRMR Between
China	1	87522	.857	.850	.049	.049	.117
	2	87077	.899	.892	.042	.047	.116
India	1	109587	.955	.953	.027	.027	.079
	2	109386	.973	.971	.021	.025	.069
Japan & Korea	1	98292	.878	.872	.037	.047	.106
	2	97997	.911	.905	.032	.041	.099
Morocco	1	73639	.814	.805	.045	.069	.163
	2	73018	.926	.921	.029	.050	.124
North America	1	195919	.824	.816	.046	.062	.141
	2	194188	.908	.901	.034	.052	.116
South Africa	1	210736	.947	.944	.023	.026	.085
	2	210397	.968	.966	.018	.022	.079
South America	1	144058	.762	.750	.050	.071	.179
	2	142532	.895	.887	.033	.049	.127
Slovakia	1	91123	.827	.818	.055	.060	.156
	2	89932	.903	.896	.042	.044	.136

Measurement invariance tests

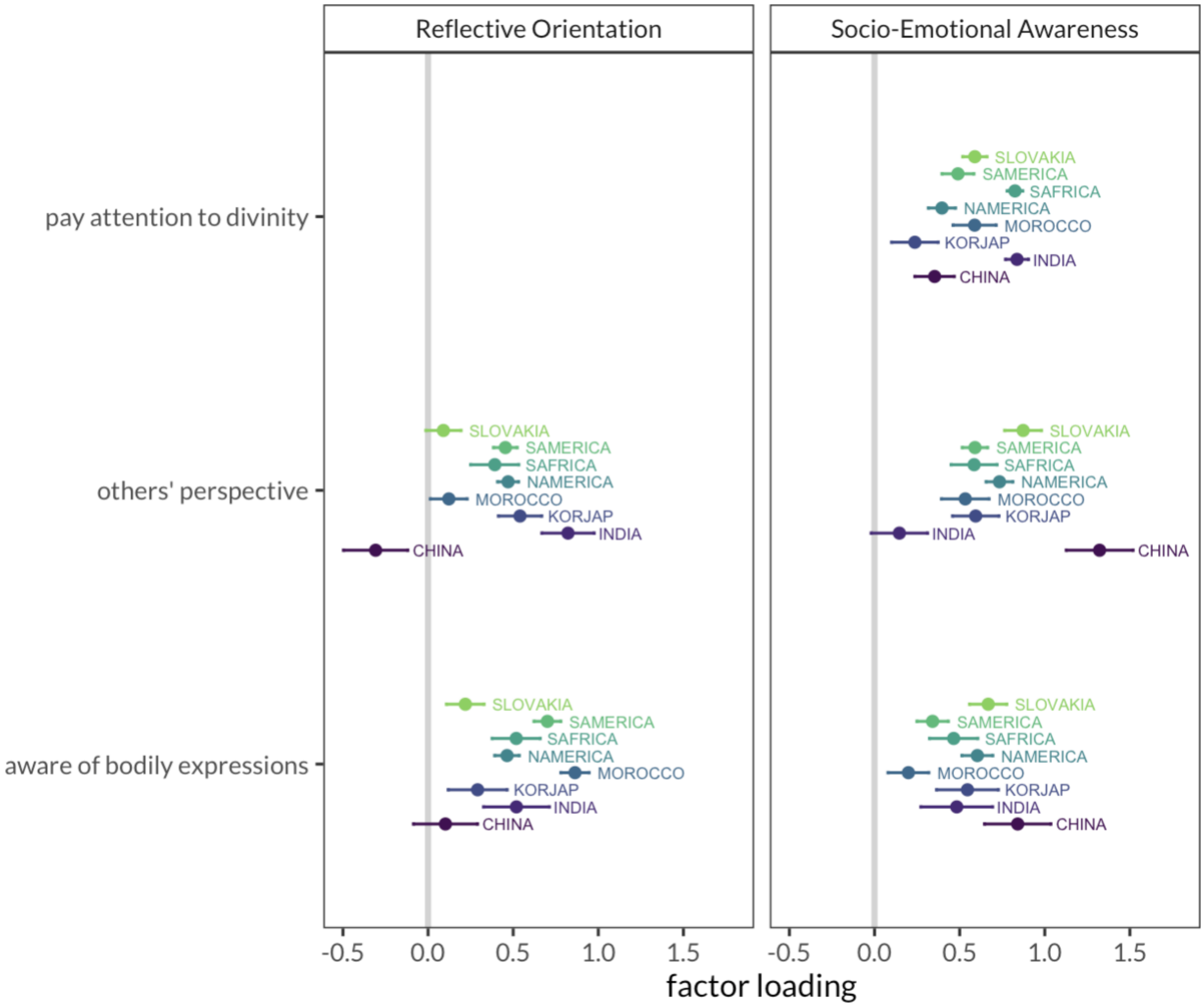

Figure S2. Non-invariant factor loadings estimated by the multiple group ML CFA constraining factor loadings across cultural regions to equality except the ones presented in the figure.

Measurement invariance of the non-isomorphic model as a robustness test. We performed an additional invariance test for non-isomorphic models (Table S19). Although the non-isomorphic model fit the data slightly better, an overall conclusion remained identical: We observed support of partial metric invariance.

Table S19. Non-isomorphic model without method factor at the between-individual level.

	BIC	CFI	Δ	TLI	Δ	RMSEA	Δ	$SRMR_{within}$	$SRMR_{Between}$
Configural	1080058	0.923		0.912		0.033		0.026	0.063
Partial metric*	1080445	0.914	.009	0.910	.002	0.033	.000	0.031	0.070
Full metric	1081210	0.904	.019	0.903	.009	0.034	.001	0.033	0.074

Note: * Loadings of three items were estimated freely across regions: (1) "pays attention to nature and divinity;" (2) "pay attention to what nature or divinity is telling them;" and (3) "aware of bodily expressions."

ML-CFA in subsets of targets. To further test the robustness of our conclusions, we repeated measurement invariance tests and sample-specific models on subsets of the targets. Although these models could not be directly compared using fit statistics (SEM models based on different samples are non-comparable), Tables S20 and S21 show the fit of the models. While excluding a *12-year-old* target decreased the model fit substantially, the factor loadings stayed virtually the same. Notably, a *12-year-old* target is not necessarily an outlier. Indeed, in the current sample of targets, young character looked different because the other targets either concern exemplars of wisdom, the self, or an average other person similar to the self. However, one arguably does not have to be an exemplar to possess *some* degree of wisdom, thus the distance between a *12-year-old* and the other targets is likely filled with other targets showing some wisdom. Moreover, there may be targets associated with unwise behavior (e.g., members of negatively stereotyped groups such as a homeless person or people suffering from addictions).

Table S20. Comparison of isomorphic ML-CFA models on subsets of targets, pooled sample.

	N	χ^2	CFI	TLI	RMSEA	$SRMR_{within}$	$SRMR_{Between}$
Full set of targets	25,288	3029.5	.956	.953	.022	.028	.082
Excluding $12_{year\text{-}old}$	20,417	3142.9	.929	.924	.025	.035	.081
Excluding 12 and $45_{year\text{-}old}$	17,998	3029.7	.927	.922	.026	.038	.081
Excluding Self	20,168	2719.7	.954	.950	.024	.029	.086
Age groups only (12, 45 and $75_{year\ old}$)	2,614	515.0	.978	.977	.022	.029	.104

Note. *N* = number of observations.

Table S21. Two-factor isomorphic model within each cultural region, including and excluding *12-year-old* target.

	N	Model	CFI	TLI	RMSEA	$SRMR_{within}$	$SRMR_{Between}$
China	2250	with 12 yo	.899	.892	.042	.047	.116
	1914	without 12yo	.852	.842	.045	.056	.142
India	2765	with 12 yo	.973	.971	.021	.025	.069
	2596	without 12yo	.971	.970	.021	.025	.067
Japan & Korea	2440	with 12 yo	.911	.905	.032	.041	.099
	2098	without 12yo	.889	.882	.034	.044	.098
Morocco	1786	with 12 yo	.926	.921	.029	.050	.124
	1462	without 12yo	.907	.901	.027	.048	.123
North America	4922	with 12 yo	.908	.901	.034	.052	.116
	4582	without 12yo	.891	.883	.035	.056	.119
South Africa	5221	with 12 yo	.968	.966	.018	.022	.079
	4878	without 12yo	.968	.965	.018	.021	.074
South America	3468	with 12 yo	.895	.887	.033	.049	.127
	3116	without 12yo	.864	.854	.035	.054	.144
Slovakia	2436	with 12 yo	.903	.896	.042	.044	.136
	1977	without 12yo	.872	.863	.043	.057	.121

Note. *N* = number of observations.

Supplementary Note B – Associations of the two dimensions of wisdom perception and the explicit ratings of wisdom

We tested associations between the two perception dimensions and the explicit ratings of wisdom. First, we included wisdom ratings into the partial metric model and estimated zero-order correlations between them. Second, we regressed latent dimensions of wisdom perception on explicit ratings of wisdom, knowledgeability, and understanding. Both first and second steps were calculated on the pooled sample and using the multiple group models. Third, we added an interaction term between the two latent dimensions of wisdom perception in predicting explicit ratings of wisdom, knowledgeability, and understanding. This last step was estimated for the single-group pooled sample model.

Correlations between dimensions of wisdom perception and explicit ratings of wisdom

Table S22 shows correlations between behavioral dimensions and ratings of wisdom, knowledgeability, and understanding on a pooled sample and for each cultural region. The differences between cultural regions were subtle.[1] In every cultural group, Reflective Orientation correlated more with wisdom than did Socio-Emotional Awareness. The two dimensions correlated with the explicit ratings of knowledgeability and understanding in a similar way they did with explicit ratings of wisdom. For the pooled sample, within-individual part, knowledgeability correlated much more with Reflective Orientation, $r = .50$, than with Socio-Emotional Awareness, $r = .21$. Understanding showed similar correlations with the two dimensions, although the difference between these correlations was smaller: $r_{\text{reflective}} = .42$ versus $r_{\text{socio-emotional}} = .33$. These observations suggest that wisdom and knowledge link mostly with Reflective Orientation, while understanding is more balanced in strengths of association between Reflective Orientation and Socio-Emotional Awareness.

In most regions, correlations of both dimensions with knowledgeability and understanding were similar. However, in North America and China, understanding showed somewhat lower correlation with Reflective Orientation than with Socio-Emotional Awareness. It suggests that in these cultural regions, attribution of knowledgeability and understanding were better differentiated when using both dimensions of wisdom perception. In Korea-Japan and Slovakia, the correlations of understanding with the two factors were almost identical, $r = .38$ and .39, .30 and .27, respectively. And in the other regions the pattern of the factors' correlations with wisdom, knowledgeability, and understanding followed the general tendency: understanding correlated with Socio-Emotional Awareness more than with Reflective Orientation, knowledgeability was closer related to Reflective Orientation than to Socio-Emotional Awareness, while attribution of wisdom showed a moderate to fair association with both latent dimensions of wisdom perception, albeit a somewhat stronger one with Reflective Orientation.

It is also noteworthy that ratings of wisdom and knowledgeability showed more consistent association with each other than with understanding (except for South Africa and Morocco). Moreover, some cultural groups (such as India and Slovakia) showed high intercorrelations of explicit ratings of wisdom, knowledge, and understanding, as well as with the

[1] Strictly speaking, the correlations cannot be compared across groups given that just the partial metric invariance was supported.

two latent wisdom dimensions. It might point to a *halo* effect, or the holistic (vs analytic) thinking tendencies in these cultural regions.

Table S22. Zero-order correlations between latent dimensions of wisdom perception and explicit attribution of wisdom, knowledge and understanding to the target.

		Pooled		CN		IND		KJ		MO		NA		SAF		SAM		SK	
		r	*p*	*r*	*p*	*r*	*p*	*r*	*p*	*r*	*p*	*r*	*p*	*r*	*p*	*r*	*p*	*r*	*p*
**Between**																			
Wise	Reflective Orientation	**.58**	*	**.36**	*	**.30**	*	**.56**	*	**.59**	*	**.35**	*	**.43**	*	**.57**	*	**.35**	*
	Socio-Emotional Awareness	**.40**	*	.14	.019	.19	.007	**.33**	*	**.30**	*	.14	*	**.35**	*	.12	.100	.05	.578
Knowledgeable	Reflective Orientation	**.63**	*	**.44**	*	**.43**	*	**.56**	*	**.64**	*	**.39**	*	**.42**	*	**.55**	*	**.34**	*
	Socio-Emotional Awareness	**.38**	*	.22	.001	.24	*	.19	.047	.22	*	.12	*	**.30**	*	-.03	.664	-.02	.830
Understanding	Reflective Orientation	**.52**	*	**.47**	*	**.32**	*	**.57**	*	**.47**	*	**.32**	*	**.35**	*	**.46**	*	.15	.052
	Socio-Emotional Awareness	**.57**	*	.28	*	**.37**	*	.06	.547	**.54**	*	.20	*	.28	*	.02	.761	.06	.519
Knowledgeable	Wise	**.65**	*	**.61**	*	**.62**	*	**.64**	*	**.67**	*	**.62**	*	**.48**	*	**.64**	*	**.66**	*
Understanding	Wise	**.45**	*	**.54**	*	**.48**	*	**.51**	*	**.47**	*	**.49**	*	**.46**	*	**.50**	*	**.47**	*
Knowledgeable	Understanding	**.46**	*	**.64**	*	**.41**	*	**.57**	*	**.43**	*	**.53**	*	**.49**	*	**.67**	*	**.41**	*
**Within**																			
Wise	Reflective Orientation	**.48**	*	**.50**	*	**.45**	*	**.50**	*	**.57**	*	**.47**	*	**.30**	*	**.56**	*	**.40**	*
	Socio-Emotional Awareness	**.30**	*	**.35**	*	.26	*	.22	*	**.32**	*	.23	*	.14	*	.20	*	.15	*
Knowledgeable	Reflective Orientation	**.54**	*	**.52**	*	**.51**	*	**.53**	*	**.61**	*	**.50**	*	**.32**	*	**.56**	*	**.43**	*
	Socio-Emotional Awareness	**.31**	*	**.36**	*	**.22**	*	.18	*	.25	*	.21	*	.14	*	.09	.001	.15	*
Understanding	Reflective Orientation	**.45**	*	**.52**	*	**.38**	*	**.49**	*	**.45**	*	**.42**	*	.28	*	**.52**	*	**.30**	*
	Socio-Emotional Awareness	**.50**	*	**.39**	*	**.39**	*	.18	*	**.53**	*	.33	*	.18	*	**.20**	*	.27	*
Knowledgeable	Wise	**.66**	*	**.62**	*	**.62**	*	**.56**	*	**.69**	*	**.61**	*	**.40**	*	**.66**	*	**.72**	*
Understanding	Wise	**.37**	*	**.61**	*	**.48**	*	**.56**	*	**.49**	*	**.49**	*	**.36**	*	**.61**	*	**.46**	*
Knowledgeable	Understanding	**.33**	*	**.62**	*	**.40**	*	**.62**	*	**.44**	*	**.48**	*	**.41**	*	**.61**	*	**.45**	*

Note. Pooled model as well as partial metric invariance model with added wisdom dimensions controlled for the target's gender. Pooled model fit: CFI = .960, TLI = .955, SRMR = .027/.081; RMSEA = .021. Multiple group model fit: CFI = .915, TLI = .909, SRMR = .026/.067, RMSEA = .032. CN – China, IND – India, KJ – Korea & Japan, MO – Morocco, NA – North America, SAF – South Africa, SAM – South America, SK – Slovakia. Bold font means significant correlations > .3.
* $p < .001$.

Predicting explicit wisdom ratings with the two dimensions of wisdom perception

We extended the pooled and multiple group models to the full structural equation models. Figure S3 shows the structure of such a model. The two latent dimensions were predictors of wisdom, knowledgeability, and understanding. At the within-individual level the latter were represented by the differences between reference target and each of the comparison targets in wisdom, knowledgeability, and understanding. At the between-individual level, these were represented by the wisdom, knowledgeability, and understanding of the reference target only.

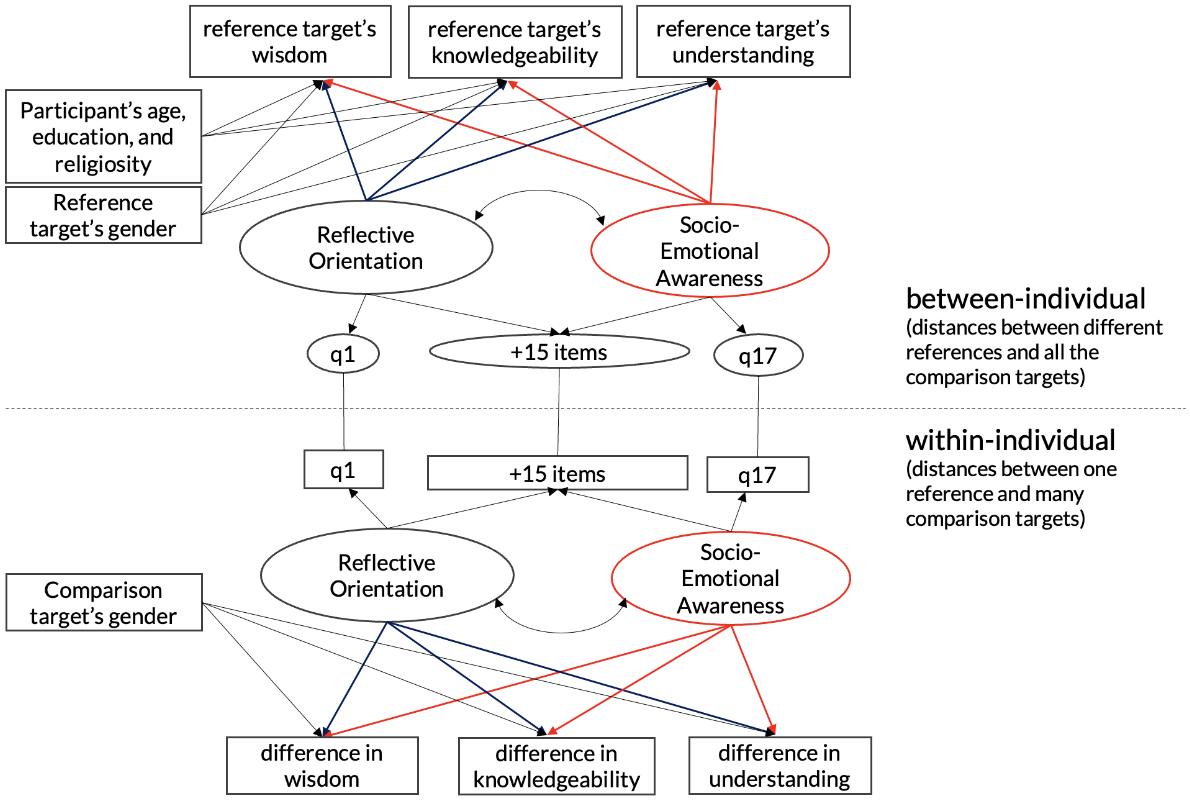

Figure S3. Two-level structural equation model. The measurement part of the model is based on a set of 17 wisdom-related characteristics (see Table S3); at the between-individual level they are represented by their intercepts. The factor loadings from two factors are constrained to be equal across the levels (isomorphic). Two factors were predictors of the explicit ratings of wisdom (as well as understanding and knowledgeability) of the targets. At the within-individual level ratings of wisdom were differences between wisdom of the reference and comparison target; wisdom of the reference target indicated wisdom at the between-individual level. Regressions of wisdom, knowledgeability, and understanding ratings on the two perception factors were controlled by the gender of the target at both levels, and by age, education, and religiosity of the participant at the between-individual level.

In these models, we simultaneously controlled for the gender of the targets (comparison target at the within-person level / reference target at the between-person level). We assigned participants' gender to the gender of "self" target. We could not control for gender directly in the multiple group model analyses, because genders were not represented for some groups (e.g., in

China all the targets were either males or gender-neutral, see Table S2). For this reason, in multiple group models we used a pseudo-continuous indicator of gender with -1 referring to male, 0 referring to gender-neutral, and 1 to female. Thereby, we were able to control for gender differences of targets across all groups. To control for individual differences in age and education, we added several control variables at the between-individual part of all the ML SEM models. We included age, parental education, and self-reported religiosity. Since religiosity measure was not available in some samples, we did not include it in the multiple group models.

Table S23 shows the regression coefficients of wisdom, knowledgeability, and understanding on the perception dimensions at the pooled sample, and Table S24 and Figure S4 show the coefficients from the multiple group model. The results of regressions differ from the zero-order correlations reported above. Reflective Orientation was still positively and significantly related to attributions of wisdom, knowledgeability, and understanding, whereas Socio-Emotional Awareness demonstrated *negative* effects on knowledgeability and wisdom and a weak positive effect on understanding. Socio-Emotional Awareness showed small and non-significant effects on wisdom in North and South Americas and Morocco. In India and South Africa, Socio-Emotional Awareness had a negative effect on understanding, and in Morocco and South America these effects were non-significant. Similar results were obtained at the between-individual level (and in the group-specific models which were easier to estimate due to lower complexity, see Table S26). Overall, the two dimensions together with control variables explained 22 and 24% of wisdom's variance at the within- and between-individual levels.

Table S23. Standardized regression coefficients of the wisdom perception dimensions predicting explicit ratings of wisdom, knowledgeability, and understanding, obtained from the pooled ML SEM model.

Independent Variables	Dependent Variables					
	Wise		Knowledgeable		Understanding	
	β	*p*	*β*	*p*	*β*	*p*
Between						
Reflective Orientation	.69	*	.82	*	.51	*
Socio-Emotional Awareness	-.46	*	-.58	*	-.28	*
Gender of targets						
Female	-.02	.209	-.04	.013	.00	.950
Not specified	-.05	.005	-.04	.042	-.05	.013
R^2	.18	*	.24	*	.11	*
Within						
Reflective Orientation	.55	*	.63	*	.38	*
Socio-Emotional Awareness	-.15	*	-.23	*	.04	.019
Gender of targets						
Female	.02	.002	.03	*	.00	.632
Not specified	-.04	*	-.07	*	-.05	*
R^2	.22	*	.28	*	.17	*

Note. Effects of participants' age, religiosity, and education are not shown. Model fit: CFI = 0.958, TLI = 0.951, SRMR = .026/.064; RMSEA = 0.020. * $p < .001$

Table S24. Standardized regression coefficients of the wisdom perception dimensions predicting explicit ratings of wisdom, knowledgeability, and understanding, obtained from the multiple group ML SEM model with partially invariant factor loadings.

		CN		IND		KJ		MO		NA		SAF		SAM		SK	
Dependent	Independent	*β*	*p*	*β*	*p*	*β*	*p*	*β*	*p*	*β*	*p*	*β*	*p*	*β*	*p*	*β*	*p*
Between																	
Wise	RO	.72	*	.88	*	.66	.001	.56	*	.59	*	.65	*	.63	*	.59	*
	SEA	-.22	.156	-.59	.001	-.42	.047	.07	.406	.03	.785	-.23	.138	-.08	.302	-.39	.003
	Gender (cont.)	-.10	.130	-.03	.580	.05	.438	-.12	.156	-.05	.360	.02	.608	-.15	.003	-.26	*
R^2		*.40*		*.24*		*.15*		*.41*		*.35*		*.20*		*.35*		*.21*	
Knowledgeable	RO	.88	*	.97	*	1.00	*	.54	*	.70	*	.82	*	.65	*	.66	*
	SEA	-.31	.014	-.62	*	-.66	.012	.04	.714	-.09	.327	-.47	.034	-.25	.001	-.50	*
	Gender (cont.)	-.15	.013	-.04	.367	<|.01|	.946	-.17	.069	-.06	.247	-.05	.281	.05	.351	-.23	.001
R^2		*.54*		*.31*		*.29*		*.42*		*.42*		*.21*		*.37*		*.24*	
Understanding	RO	.16	.192	.90	*	-.03	.871	.71	*	.21	.008	.52	.003	.53	*	.21	.058
	SEA	.44	*	-.49	.003	.38	.053	-.17	.071	.46	*	-.20	.234	-.16	.030	-.09	.501
	Gender (cont.)	-.08	.234	.02	.653	<|.01|	.969	.15	.097	<|.01|	.990	-.04	.334	.06	.255	-.23	.003
R^2		*.35*		*.30*		*.13*		*.40*		*.36*		*.13*		*.25*		*.07*	
Within																	
Wise	RO	.73	*	.84	*	.59	*	.45	*	.57	*	.56	*	.59	*	.69	*
	SEA	-.28	*	-.38	.001	-.19	.002	.04	.299	<|.01|	.980	-.32	*	-.02	.466	-.39	*
	Gender (cont.)	.10	*	-.02	.389	-.12	*	.12	*	.01	.345	.09	*	.06	*	.02	.237
R^2		*.31*		*.29*		*.21*		*.26*		*.33*		*.14*		*.33*		*.22*	
Knowledgeable	RO	.86	*	.89	*	.81	*	.53	*	.69	*	.66	*	.63	*	.77	*
	SEA	-.39	*	-.41	*	-.40	*	-.02	.669	-.15	*	-.40	*	-.15	*	-.45	*
	Gender (cont.)	.14	*	.03	.179	-.06	.010	.08	.003	-.03	.027	.08	*	-.01	.680	.10	*
R^2		*.41*		*.32*		*.32*		*.31*		*.39*		*.17*		*.35*		*.27*	
Understanding	RO	.09	.146	.78	*	.18	.001	.46	*	.22	*	.42	*	.53	*	.22	*
	SEA	.45	*	-.29	.003	.24	*	<|.01|	.918	.42	*	-.17	.007	-.01	.802	.11	.014
	Gender (cont.)	.08	*	-.04	.013	-.12	*	.09	*	-.06	*	.07	*	-.04	.008	.13	*
R^2		*.29*		*.29*		*.17*		*.24*		*.33*		*.10*		*.28*		*.13*	

Note. RO = Reflection Orientation. SEA = Socio-Emotional Awareness. Based on multiple-group partial metric invariance model of wisdom perception. The effects of participants' age, religiosity, and education are not shown. Multiple group model fit: CFI = 0.914, TLI = 0.905, SRMR = .031/.061; RMSEA = 0.031. CN – China, IND – India, KJ – Korea & Japan, MO – Morocco, NA – North America, SAF – South Africa, SAM – South America, SK – Slovakia.
* $p < .001$.

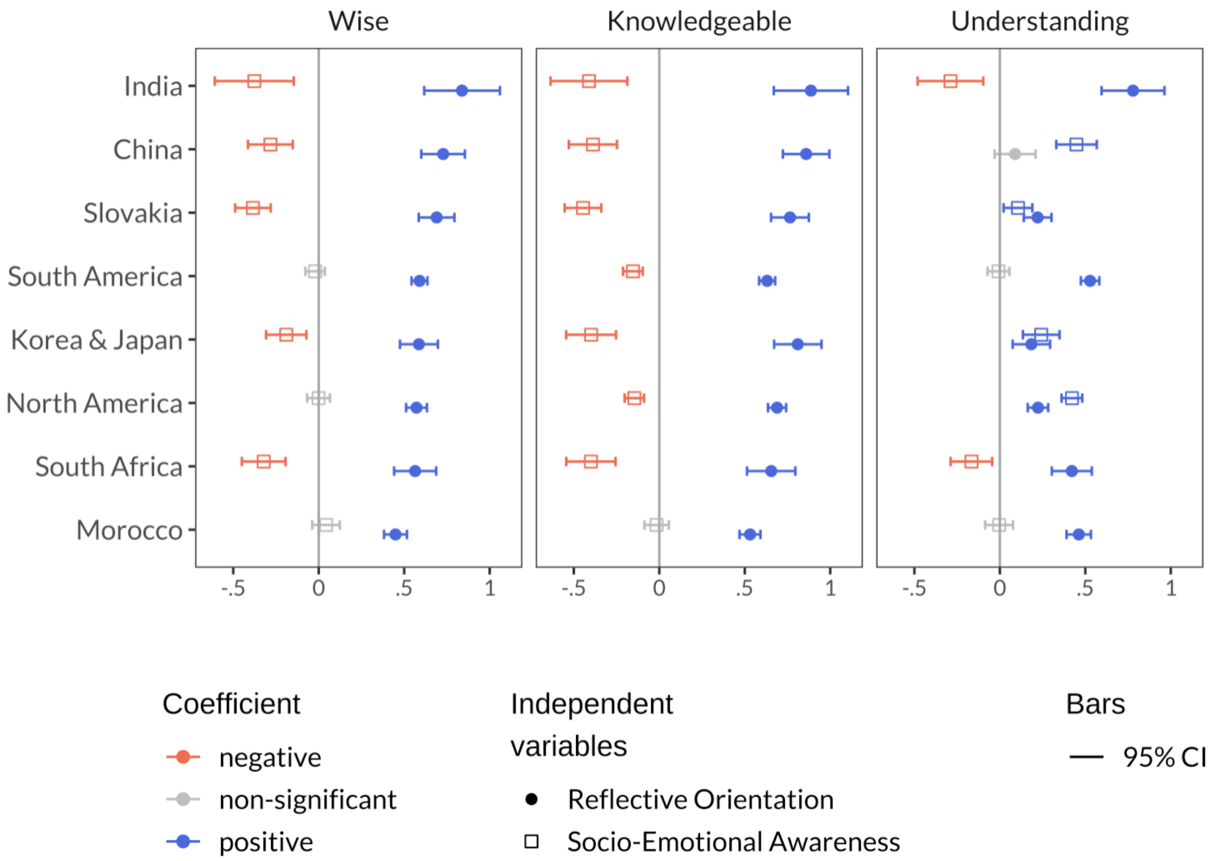

Figure S4. Standardized regression coefficients estimated by the ML SEM model in which the two dimensions of wisdom perception predicted explicit ratings of wisdom, knowledgeability, and understanding of targets. Vertical line represents zero. Horizontal bars represent 95% confidence intervals. “Significant” stands for 95% CI including zero.

Interactions between the two dimensions of wisdom perception in predicting explicit ratings of wisdom, knowledgeability, and understanding

Table S25. Regression coefficients from the ML SEM model at the pooled sample with an interaction term between the two latent variables – dimensions of wisdom perception, Bayesian estimation.

	Wise [95% CI]			Knowledgeable [95% CI]			Understanding [95% CI]		
Between									
Reflective Orientation	.70	[.61	.79]	.81	[.72	.91]	.52	[.43	.62]
Socio-Emotional Awareness	-.43	[-.53	-.34]	-.56	[-.66	-.47]	-.26	[-.35	-.16]
RO x SEA Interaction	.07	[.04	.10]	.03	[.001	.06]	.05	[.02	.08]
Target gender - female	-.05	[-.09	-.01]	-.06	[-.09	-.02]	-.02	[-.06	.02]
Target gender – not specified	-.07	[-.11	-.03]	-.04	[-.08	-.001]	-.06	[-.10	-.02]
Within									
Reflective Orientation	.51	[.48	.53]	.59	[.56	.61]	.33	[.30	.35]
Socio-Emotional Awareness	-.15	[-.18	-.12]	-.23	[-.27	-.21]	.05	[.02	.08]
RO x SEA Interaction	.09	[.08	.10]	.10	[.09	.11]	.09	[.08	.11]
Target gender - female	.02	[.01	.04]	.03	[.02	.05]	.001	[-.01	.02]
Target gender – not specified	-.04	[-.06	-.03]	-.08	[-.09	-.06]	-.06	[-.07	-.04]

Note. RO = Reflection Orientation. SEA = Socio-emotional Awareness. 95% CI – Bayesian credible intervals. Controlled for parental education and personal religiosity. The regression coefficients are standardized, estimated by the Bayesian model, see traceplots and other convergence information in OSF directory https://osf.io/m4dxv.

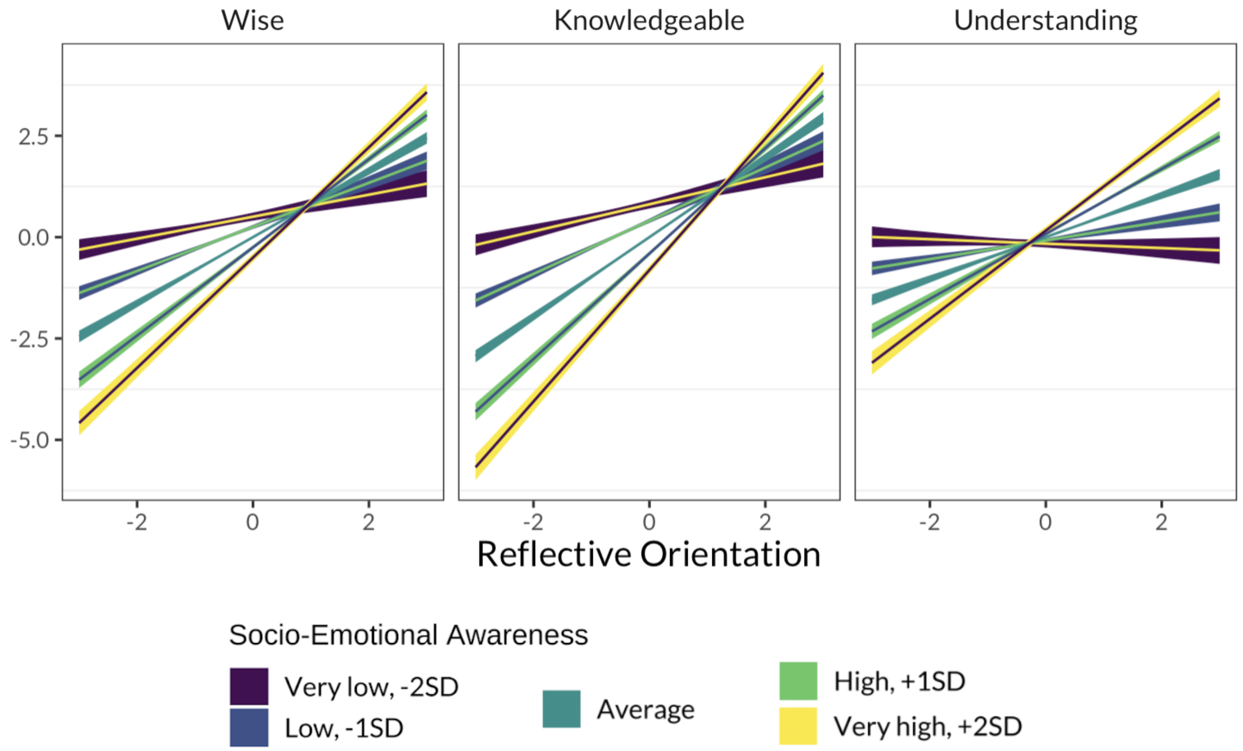

Figure S5. Differences between interactive effects in predicting wisdom, knowledgeability, and understanding with Reflective Orientation and Socio-Emotional Awareness at the within level. *Note.* The height of the ribbons is 95% credible interval; SD is the standard deviation.

Multiple group model was not estimable due to high complexity of the latent variable interactions modeling; therefore, we fitted the ML SEM with interactions within each cultural region separately (Table S26). Models fitted within each of the cultural groups revealed similar pattern of results as the pooled model at the within level: Effects of Reflective Orientation were positive, strong, and stable across models and regions; effects of Socio-Emotional Awareness varied across regions, but in general tended to be negative for wisdom and knowledgeability and positive for understanding (with exception of India and South Africa). Interaction terms were positive and significant in most cases: more pronounced for knowledgeability and understanding, but less so for wisdom. Among cultural groups, Morocco showed the least number of significant effects; under most conditions all effects except the one for Reflective Orientation were close to zero. In contrast, models fit in North America and China showed the clearest differentiation between prediction of wisdom and knowledgeability on one hand and understanding on the other. At the between-individual level, the results replicated the ones at the within-individual level, albeit showed larger coefficients and larger standard errors.

Table S26. Cross-regional consistency of effects on ratings of wisdom, knowledgeability, and understanding is higher for Reflective Orientation as compared to Socio-Emotional Awareness.

		CN		IND		KJ		MO		NA		SAF		SAM		SK	
		b	*p*	*b*	*p*	*b*	*p*	*b*	*p*	*b*	*p*	*b*	*p*	*b*	*p*	*b*	*p*
Between																	
Wise	RO	2.04	*	2.03	*	1.93	.028	1.52	*	1.26	*	1.29	*	1.42	*	1.42	*
	SEA	-0.71	.026	-1.51	*	-1.30	.081	0.16	.368	0.16	.215	-0.27	.228	0.17	.269	-0.85	.003
	RO x SEA	0.57	.032	-0.23	.149	0.01	.482	0.57	.231	-0.47	.043	-0.09	.255	-1.00	*	-0.31	.245
Knowledgeable	RO	2.92	*	2.31	*	3.19	.001	1.29	*	1.51	*	1.72	*	1.34	*	1.59	*
	SEA	-1.11	.004	-1.63	*	-2.19	.011	0.27	.274	-0.03	.437	-0.83	.008	-0.09	.377	-1.26	*
	RO x SEA	0.85	.006	-0.46	.013	-0.17	.181	0.91	.096	-0.79	.003	-0.16	.112	-1.61	*	-1.21	.011
Understanding	RO	0.61	.054	2.19	*	-0.72	.252	1.62	*	0.60	.001	0.77	.025	1.07	*	0.55	.041
	SEA	1.17	.001	-1.30	*	1.33	.085	-0.61	.102	1.06	*	-0.07	.420	-0.01	.495	-0.23	.238
	RO x SEA	0.56	.044	-0.34	.049	-0.06	.368	-0.20	.398	0.08	.390	-0.02	.432	-1.49	*	0.14	.395
Within																	
Wise	RO	1.06	*	1.29	*	0.70	*	0.78	*	0.71	*	0.85	*	0.84	*	0.67	*
	SEA	-0.43	*	-0.70	*	-0.13	.027	0.07	.186	0.05	.130	-0.47	*	0.04	.209	-0.47	*
	RO x SEA	0.08	.045	0.32	*	0.09	.010	0.07	.229	0.21	*	0.28	*	0.23	*	0.22	*
Knowledgeable	RO	1.51	*	1.42	*	1.10	*	0.87	*	0.90	*	0.97	*	0.84	*	0.80	*
	SEA	-0.71	*	-0.79	*	-0.40	*	-0.04	.299	-0.18	*	-0.58	*	-0.10	.021	-0.59	*
	RO x SEA	0.11	.021	0.31	*	0.19	*	0.22	.004	0.27	*	0.29	*	0.25	*	0.27	*
Understanding	RO	0.01	.453	1.18	*	0.20	.015	0.73	*	0.24	*	0.55	*	0.70	*	0.13	.004
	SEA	0.69	*	-0.54	*	0.36	*	0.01	.466	0.63	*	-0.15	.065	0.07	.044	0.09	.018
	RO x SEA	0.13	.006	0.31	*	0.10	.008	0.25	.003	0.20	*	0.32	*	0.22	*	0.14	*

Note: Unstandardized regression coefficients from the ML SEM model with an interaction term between the two latent variables – dimensions of wisdom perception, fitted in each region separately. Models were fitted with Bayesian estimation. CN – China, IND – India, KJ – Korea & Japan, MO – Morocco, NA – North America, SAF – South Africa, SAM – South America, SK – Slovakia. *b* = unstandardized regression coefficient, *p* = p-value.

* $p < .001$.

Robustness checks of regressions of ratings of wisdom, knowledgeability and understanding on the two dimensions of wisdom perception

Since the two dimensions of wisdom perception were positively correlated, we ran a series of robustness checks. The first test was fitting the regularized (lasso) models to check if the two factors, and their interaction, uniquely contributed to the ratings of wisdom. Furthermore, we used different subsets of targets. An outlier target (characterized by unique qualities, e.g., extremely wise and extremely low on both dimensions of wisdom perception) might have biased the results, exaggerating or attenuating the association between the explicit ratings of wisdom and the two dimensions of wisdom perception. As is apparent from the next section, one target, namely the *12-year-old*, stood out from the list of targets. We tested the same model against subsamples excluding the *12-year-old* (M1), excluding all three age groups (M2), including age groups only (M3), five targets defined by their occupation only (M4), and two ways to random selection of targets: In the first (M5), targets were randomly selected within each individual independently (pseudo-randomization of stimuli targets). Another approach to random selection is to select targets randomly for all individuals (M6).

Results in Table S27 show different inclusions of targets (M1-M6) only slightly changed the main effects of Reflective Orientation and Socio-Emotional Awareness on wisdom. These coefficients were the lowest when the sample excluded the *12-year-old* target. Yet in every subset of targets the effects were highly significant and large in size. In all of them, the coefficients were quite similar to the ones obtained on the full sample, showing that the outlier targets did not bias the results.

The main effects of Reflective Orientation and Socio-Emotional Awareness showed the same tendency with regard to knowledgeability, and Reflective Orientation showed similarly stable tendencies with regard to understanding. However, the positive association between Socio-Emotional Awareness and understanding varied across different subsets of targets. It was substantial when all the targets were included, when only professional targets were included, and when the *12-year-old* or all the age-defined targets were excluded from analyses. However, it was negligible when only the age-defined targets were included (M3) and when the subsets of targets were chosen at random for analyses (M5-M6). This finding suggests that the effect of Socio-Emotional Awareness on ratings of understanding is unstable and depends on the specific set of targets in the study. Interestingly, it seems to come from a combination of targets defined by their occupation.

Unlike the main effects, the interaction effect was less stable when performing robustness checks. In most analyses (including penalized lasso regression on factor scores) it was not significant; in some subsamples it switched the sign. Dropping the *12-year-old* and other age-defined targets (M1-M2) as well as limiting the target list to professional groups led the interaction term to switch sign to negative. If we are to unpack the latter interaction results, then it should go as follows: To be considered wise, a target should be higher on Reflective Orientation and lower on Socio-Emotional Awareness. The difference from the main analysis is that here, among the targets higher on Socio-Emotional Awareness, the importance of Reflective Orientation decreases (while it increased when the *12-year-old* was retained among the targets in the main analysis). For example, the *politician*, *scientist*, and *12-year-old* were the lowest on Socio-Emotional Awareness. Reflective Orientation was important when comparing a *12-year-old* and a *politician* on wisdom, but less when comparing a *politician* and a *scientist*.

Together, these results suggest that the main effects are robust while the interaction terms are less so, because interaction terms switched signs and varied in their magnitude across different subsets of targets.

Table S27. Standardized regression coefficients estimated by a series of Lasso regressions with predicted factor scores of the two wisdom perception dimensions as predictors.

	M0	M1	M2	M3	M4	M5	M6
	All targets	No 12yo group	No age groups	Age groups only	Professions only	Randomly selected targets within individual	Random selection of targets for all individuals
$r_{dimensions}$	0.79	0.73	0.75	0.86	0.72	0.80	0.85
Wise							
Reflective Orientation	0.98*	0.84*	0.84*	1.11*	0.86*	1.00*	0.94*
Socio-Emotional Awareness	-0.30*	-0.27*	-0.27*	-0.36*	-0.22*	-0.37*	-0.28*
Interaction	0.01	-0.11	-0.11	0.03	-0.12	0.02	0.02
	p=.901	*p*=.003	*p*=.001	*p*=.706	*p*=.008	*p*=.746	*p*=.889
Lambda	0.00096	0.00066	0.00065	0.00098	0.00072	0.00093	0.00076
Knowledgeable							
Rational	1.19*	1.11*	1.07*	1.12*	1.14*	1.22*	1.03*
Socio-Emotional Awareness	-0.49*	-0.54*	-0.52*	-0.38*	-0.48*	-0.51*	-0.35*
Interaction	0.06	-0.10	-0.11	0.08	-0.14	0.07	0.06
	p=.014	*p*=.005	*p*=.001	*p*=.031	*p*=.002	*p*=.061	*p*=.047
Lambda	0.0011	0.00081	7e-04	0.00092	0.00082	0.0011	0.00091
Understanding							
Rational	0.63*	0.47*	0.48*	0.80*	0.55*	0.60*	0.72*
Socio-Emotional Awareness	0.12*	0.17*	0.15*	0.00	0.15	0.15	0.06
				p=1.00	*p*=.003	*p*=.006	*p*=.447
Interaction	0.00	-0.09	-0.11*	0.01	-0.11	0.00	0.02
	p=1.000	*p*=.013		*p*=1.000	*p*=.019	*p*=1.000	*p*=.977
Lambda	0.0022	0.0013	0.0013	0.0033	0.0014	0.0022	0.0027

Note. We estimated confidence intervals via bootstrapping. Lasso regressions involve an arbitrarily set penalization parameter lambda; in order to avoid arbitrariness we used cross-validation technique to determine an optimal lambda for each model. The models describe the within-individual level estimated at the pooled sample. $r_{dimensions}$ – correlation between factor scores of Reflective Orientation and Socio-emotional awareness in each subsample. $^{*}p < .001$.

Figures S6-S7 provide further insights on the nature of the main and interaction effects. Figure S7 flips the moderator and the main effect roles of the two dimensions: at the within level,

Socio-Emotional Awareness is negatively related to wisdom ratings except when the Reflective Orientation is the highest, in the latter case the association between Socio-Emotional Awareness and attribution of wisdom appears weak or even positive.

At the between level, the association between Socio-Emotional Awareness and attribution of wisdom remained strongly negative at all levels of Reflective Orientation, though somewhat less pronounced at the higher levels. Figure S8 shows interaction plots for the ratings of wisdom, knowledgeability, and understanding estimated at the between level – unlike within-person level, the regression lines do not cross here. This observation implies that, first, Reflective Orientation is strongly and positively associated with the attribution of epistemic qualities (wisdom, knowledge, understanding), and second, that Socio-Emotional Awareness has negative association with wisdom and knowledgeability at all levels of Reflective Orientation. Notably, for attribution of understanding, the effect is similar except the interaction also leads to convergence of lines at the higher levels of Reflective Orientation.

Finally, Figure S8 provides a sanity check of our interpretations of the main and interactions terms. For example, columns in the upper left panel show the number of participants who assigned the lowest level of Reflective Orientation to different targets – e.g., only 31 out of 670 participants in the lowest quartile of Reflective Orientation assigned the lowest Socio-Emotional Awareness to themselves, whereas 142 participants rated *12-year-old* this way. Red line in this panel shows that within the first and the second quartiles of Reflective Orientation, the lower quartile of Socio-Emotional Awareness had the highest ratings of wisdom. Overall, the plot confirms the negative effect of Socio-Emotional Awareness on the ratings of wisdom when Reflective Orientation is at its mid- or low-level.

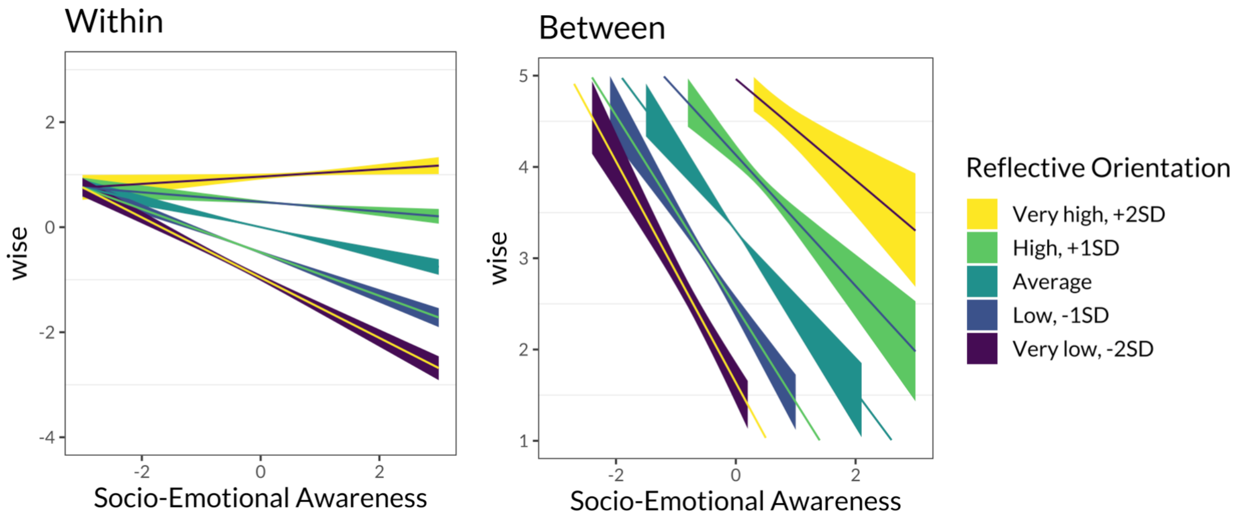

Figure S6. Interaction plot where Reflective Orientation is treated as a moderator of the effect of Socio-Emotional Awareness on attribution of wisdom.

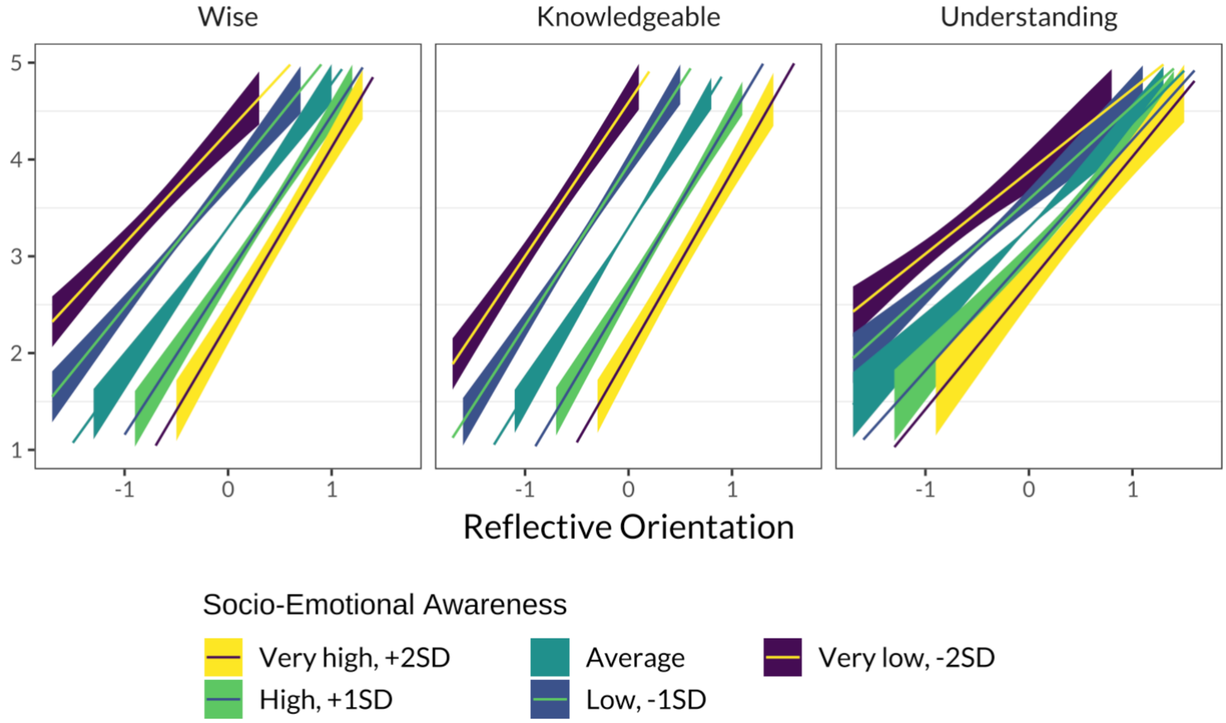

Figure S7. Interaction plot for the between-level terms.

Table S28. Multilevel pairwise correlations between each indicator of wisdom perception and explicit ratings of wisdom.

<table>
<tr><th></th><th>CN</th><th>IN</th><th>KJ</th><th>MO</th><th>NA</th><th>SAf</th><th>SAm</th><th>SK</th></tr>
<tr><td>**Between-person**</td><td></td><td></td><td></td><td></td><td></td><td></td><td></td><td></td></tr>
<tr><td>*Reflective Orientation items*</td><td></td><td></td><td></td><td></td><td></td><td></td><td></td><td></td></tr>
<tr><td>Q5 think logically</td><td>**.66**</td><td>**.55**</td><td>**.51**</td><td>**.69**</td><td>**.66**</td><td>**.47**</td><td>**.57**</td><td>**.52**</td></tr>
<tr><td>Q2 think in many ways</td><td>**.68**</td><td>**.54**</td><td>**.47**</td><td>**.67**</td><td>**.66**</td><td>**.47**</td><td>**.61**</td><td>**.43**</td></tr>
<tr><td>Q6 apply experiences</td><td>**.41**</td><td>**.54**</td><td>**.49**</td><td>**.69**</td><td>**.67**</td><td>**.51**</td><td>**.65**</td><td>**.45**</td></tr>
<tr><td>Q7 control of emotions</td><td>**.60**</td><td>**.57**</td><td>**.43**</td><td>**.59**</td><td>**.65**</td><td>**.44**</td><td>**.58**</td><td>**.42**</td></tr>
<tr><td>Q11 recognize change</td><td>**.68**</td><td>**.47**</td><td>**.47**</td><td>**.60**</td><td>**.64**</td><td>**.46**</td><td>**.50**</td><td>.29</td></tr>
<tr><td>Q13 hide emotions</td><td>**.64**</td><td>**.37**</td><td>**.33**</td><td>.29</td><td>**.43**</td><td>**.39**</td><td>**.40**</td><td>.29</td></tr>
<tr><td>Q14 think before acting</td><td>**.69**</td><td>**.52**</td><td>**.44**</td><td>**.72**</td><td>**.66**</td><td>**.51**</td><td>**.65**</td><td>**.47**</td></tr>
<tr><td>Q9 benefit for their group</td><td>**.22**</td><td>.04</td><td>.10</td><td>**.34**</td><td>.20</td><td>**.37**</td><td>**.30**</td><td>.04</td></tr>
<tr><td>*Socio-Emotional Awareness Items*</td><td></td><td></td><td></td><td></td><td></td><td></td><td></td><td></td></tr>
<tr><td>Q4 (intellectual) humility</td><td>**.49**</td><td>.26</td><td>**.37**</td><td>**.38**</td><td>**.42**</td><td>**.40**</td><td>.22</td><td>.12</td></tr>
<tr><td>Q15 pay attention to divinity</td><td>**.41**</td><td>**.30**</td><td><|.01|</td><td>.14</td><td>**.25**</td><td>**.30**</td><td>-.03</td><td>-.20</td></tr>
<tr><td>Q10 others' perspective</td><td>**.44**</td><td>**.48**</td><td>**.35**</td><td>.03</td><td>**.55**</td><td>**.44**</td><td>**.40**</td><td>.17</td></tr>
<tr><td>Q18 aware of bodily expressions</td><td>**.41**</td><td>**.47**</td><td>**.33**</td><td>**.59**</td><td>**.50**</td><td>**.48**</td><td>**.45**</td><td>.18</td></tr>
<tr><td>Q3 care for others' feelings</td><td>**.38**</td><td>**.23**</td><td>**.38**</td><td>**.39**</td><td>**.33**</td><td>**.34**</td><td>.16</td><td>.28</td></tr>
<tr><td>Q12 neutral advice</td><td>**.46**</td><td>-.04</td><td>.15</td><td>-.14</td><td>.28</td><td>**.32**</td><td>.24</td><td>-.29</td></tr>
<tr><td>Q17 pay attention to emotions</td><td>**.42**</td><td>**.33**</td><td>.18</td><td>.01</td><td>.27</td><td>**.40**</td><td>.12</td><td>-.08</td></tr>
<tr><td>Q8 sense of humor</td><td>**.34**</td><td>.08</td><td>.20</td><td><|.01|</td><td>.19</td><td>.20</td><td>-.03</td><td>.01</td></tr>
<tr><td>**Within-person**</td><td></td><td></td><td></td><td></td><td></td><td></td><td></td><td></td></tr>
<tr><td>*Reflective Orientation items*</td><td></td><td></td><td></td><td></td><td></td><td></td><td></td><td></td></tr>
<tr><td>Q5 think logically</td><td>**.43**</td><td>**.40**</td><td>**.39**</td><td>**.39**</td><td>**.45**</td><td>.20</td><td>**.43**</td><td>**.37**</td></tr>
<tr><td>Q2 think in many ways</td><td>**.41**</td><td>**.37**</td><td>**.33**</td><td>**.36**</td><td>**.42**</td><td>.17</td><td>**.39**</td><td>**.34**</td></tr>
<tr><td>Q6 apply experiences</td><td>.23</td><td>**.35**</td><td>**.32**</td><td>**.39**</td><td>**.46**</td><td>.22</td><td>**.43**</td><td>**.34**</td></tr>
<tr><td>Q7 control of emotions</td><td>**.37**</td><td>**.33**</td><td>**.25**</td><td>**.36**</td><td>**.41**</td><td>.19</td><td>**.34**</td><td>**.32**</td></tr>
<tr><td>Q11 recognize change</td><td>**.41**</td><td>**.36**</td><td>**.33**</td><td>**.35**</td><td>**.41**</td><td>.17</td><td>**.34**</td><td>**.24**</td></tr>
<tr><td>Q13 hide emotions</td><td>**.31**</td><td>**.22**</td><td>.18</td><td>.15</td><td>.18</td><td>.11</td><td>.16</td><td>**.23**</td></tr>
<tr><td>Q14 think before acting</td><td>**.37**</td><td>**.34**</td><td>**.33**</td><td>**.39**</td><td>**.38**</td><td>.20</td><td>**.38**</td><td>**.32**</td></tr>
<tr><td>Q9 benefit for their group</td><td>.01</td><td>.08</td><td>-.09</td><td>.16</td><td>.07</td><td>.09</td><td>.13</td><td>.07</td></tr>
<tr><td>*Socio-Emotional Awareness Items*</td><td></td><td></td><td></td><td></td><td></td><td></td><td></td><td></td></tr>
<tr><td>Q4 (intellectual) humility</td><td>.23</td><td>.23</td><td>.21</td><td>.20</td><td>.27</td><td>.09</td><td>.17</td><td>.16</td></tr>
<tr><td>Q15 pay attention to divinity</td><td>.15</td><td>.22</td><td>.01</td><td>.14</td><td>.11</td><td>.09</td><td>.02</td><td>.07</td></tr>
<tr><td>Q10 others' perspective</td><td>.21</td><td>**.34**</td><td>**.30**</td><td>-.03</td><td>**.35**</td><td>.14</td><td>.26</td><td>.15</td></tr>
<tr><td>Q18 aware of bodily expressions</td><td>.26</td><td>**.35**</td><td>.15</td><td>**.32**</td><td>**.30**</td><td>.16</td><td>.27</td><td>.16</td></tr>
<tr><td>Q3 care for others' feelings</td><td>.16</td><td>.25</td><td>.25</td><td>.21</td><td>.21</td><td>.09</td><td>.13</td><td>.20</td></tr>
<tr><td>Q12 neutral advice</td><td>.23</td><td>.09</td><td>.11</td><td>-.06</td><td>.15</td><td>.04</td><td>.11</td><td>-.07</td></tr>
<tr><td>Q17 pay attention to emotions</td><td>.22</td><td>.22</td><td>.06</td><td><|.01|</td><td>.11</td><td>.08</td><td>.12</td><td>-.01</td></tr>
<tr><td>Q8 sense of humor</td><td>.20</td><td>.13</td><td>.09</td><td>.01</td><td>.11</td><td><|.01|</td><td>.03</td><td>.06</td></tr>
</table>

Note: CN = China, IN = India, KJ = Korea and Japan, MO = Morocco, NA = North America (US and Canada), SAf = South Africa, Sam = South American, SK = Slovakia. Correlations > .3 in magnitude are in bold, negative correlations are in red font.

Table S29. Predicting explicit ratings of wisdom with wisdom perception indicators (rather than dimensions) at each cultural region, standardized regression coefficients.

<table>
<tr><th></th><th colspan="2">China</th><th colspan="2">India</th><th colspan="2">Korea & Japan</th><th colspan="2">Morocco</th><th colspan="2">North America</th><th colspan="2">South Africa</th><th colspan="2">South America</th><th colspan="2">Slovakia</th></tr>
<tr><th></th><th>b</th><th>p</th><th>b</th><th>p</th><th>b</th><th>p</th><th>b</th><th>p</th><th>b</th><th>p</th><th>b</th><th>p</th><th>b</th><th>p</th><th>b</th><th>p</th></tr>
<tr><td colspan="17">Reflective Orientation items</td></tr>
<tr><td>Q5 think logically</td><td>.21</td><td>*</td><td>.15</td><td>*</td><td>.24</td><td>*</td><td>.11</td><td>*</td><td>.17</td><td>*</td><td>.09</td><td>*</td><td>.19</td><td>*</td><td>.13</td><td>*</td></tr>
<tr><td>Q2 think in many ways</td><td>.13</td><td>*</td><td>.09</td><td>*</td><td>.08</td><td>.010</td><td>.07</td><td>.009</td><td>.11</td><td>*</td><td>.05</td><td>.004</td><td>.13</td><td>*</td><td>.07</td><td>.022</td></tr>
<tr><td>Q6 apply experiences</td><td>.02</td><td>.354</td><td>.08</td><td>*</td><td>.13</td><td>*</td><td>.15</td><td>*</td><td>.25</td><td>*</td><td>.12</td><td>*</td><td>.22</td><td>*</td><td>.17</td><td>*</td></tr>
<tr><td>Q7 control of emotions</td><td>.13</td><td>*</td><td>.05</td><td>.011</td><td><|.01|</td><td>.991</td><td>.11</td><td>*</td><td>.11</td><td>*</td><td>.07</td><td>*</td><td>.07</td><td>*</td><td>.06</td><td>.014</td></tr>
<tr><td>Q11 recognize change</td><td>.14</td><td>*</td><td>.09</td><td>*</td><td>.10</td><td>*</td><td>.09</td><td>.001</td><td>.10</td><td>*</td><td>.06</td><td>.001</td><td>.07</td><td>.001</td><td>.05</td><td>.063</td></tr>
<tr><td>Q13 hide emotions</td><td>.04</td><td>.126</td><td>.04</td><td>.033</td><td>-.01</td><td>.708</td><td><|.01|</td><td>1.000</td><td>.07</td><td>*</td><td>.04</td><td>.009</td><td>.03</td><td>.208</td><td>.04</td><td>.152</td></tr>
<tr><td>Q14 think before acting</td><td>.03</td><td>.251</td><td>.03</td><td>.154</td><td>.11</td><td>.001</td><td>.09</td><td>.001</td><td>.03</td><td>.182</td><td>.07</td><td>.001</td><td>.07</td><td>.002</td><td>.07</td><td>.018</td></tr>
<tr><td>Q9 benefit for their group</td><td>-.08</td><td>*</td><td>-.02</td><td>.241</td><td>-.07</td><td>.005</td><td>.05</td><td>.027</td><td>-.06</td><td>.001</td><td><|.01|</td><td>.624</td><td>-.01</td><td>.523</td><td>-.04</td><td>.055</td></tr>
<tr><td colspan="17">Socio-Emotional Awareness Items</td></tr>
<tr><td>Q4 (intellectual) humility</td><td>-.04</td><td>.136</td><td><|.01|</td><td>.731</td><td>.03</td><td>.321</td><td>.06</td><td>.010</td><td>.08</td><td>*</td><td><|.01|</td><td>.703</td><td>.08</td><td>*</td><td><|.01|</td><td>.756</td></tr>
<tr><td>Q15 pay attention to divinity</td><td>.04</td><td>.035</td><td>-.01</td><td>.591</td><td>-.01</td><td>.688</td><td>.03</td><td>.278</td><td>.04</td><td>.021</td><td>-.01</td><td>.501</td><td>-.02</td><td>.210</td><td>-.03</td><td>.181</td></tr>
<tr><td>Q10 others' perspective</td><td>-.01</td><td>.684</td><td>.09</td><td>*</td><td>.04</td><td>.207</td><td>-.10</td><td>*</td><td>.06</td><td>.017</td><td>.02</td><td>.235</td><td>.03</td><td>.155</td><td>-.03</td><td>.289</td></tr>
<tr><td>Q18 aware of bodily expressions</td><td>.06</td><td>.029</td><td>.11</td><td>*</td><td><|.01|</td><td>.715</td><td>.03</td><td>.320</td><td><|.01|</td><td>.895</td><td>.05</td><td>.008</td><td>.01</td><td>.479</td><td>.03</td><td>.395</td></tr>
<tr><td>Q3 care for others' feelings</td><td>-.05</td><td>.080</td><td>.01</td><td>.605</td><td>.04</td><td>.149</td><td>.07</td><td>.004</td><td>.03</td><td>.215</td><td>-.03</td><td>.096</td><td><|.01|</td><td>.965</td><td>.04</td><td>.262</td></tr>
<tr><td>Q12 neutral advice</td><td>.01</td><td>.644</td><td>-.03</td><td>.115</td><td>-.06</td><td>.030</td><td>-.03</td><td>.178</td><td>-.02</td><td>.313</td><td>-.03</td><td>.128</td><td>-.04</td><td>.031</td><td>-.15</td><td>*</td></tr>
<tr><td>Q17 pay attention to emotions</td><td><|.01|</td><td>.867</td><td>-.02</td><td>.381</td><td>-.06</td><td>.026</td><td><|.01|</td><td>.748</td><td>-.03</td><td>.145</td><td>-.02</td><td>.286</td><td>.01</td><td>.614</td><td>-.11</td><td>*</td></tr>
<tr><td>Q8 sense of humor</td><td><|.01|</td><td>.902</td><td>-.02</td><td>.374</td><td>.03</td><td>.388</td><td>-.02</td><td>.370</td><td><|.01|</td><td>.782</td><td>-.05</td><td>.023</td><td>-.03</td><td>.161</td><td>-.09</td><td>*</td></tr>
</table>

Note: Calculated with mixed linear regressions of the within-person wisdom attribution (dependent variable). *b* = unstandardized regression coefficient, *p* = p-value. * $p < .001$.

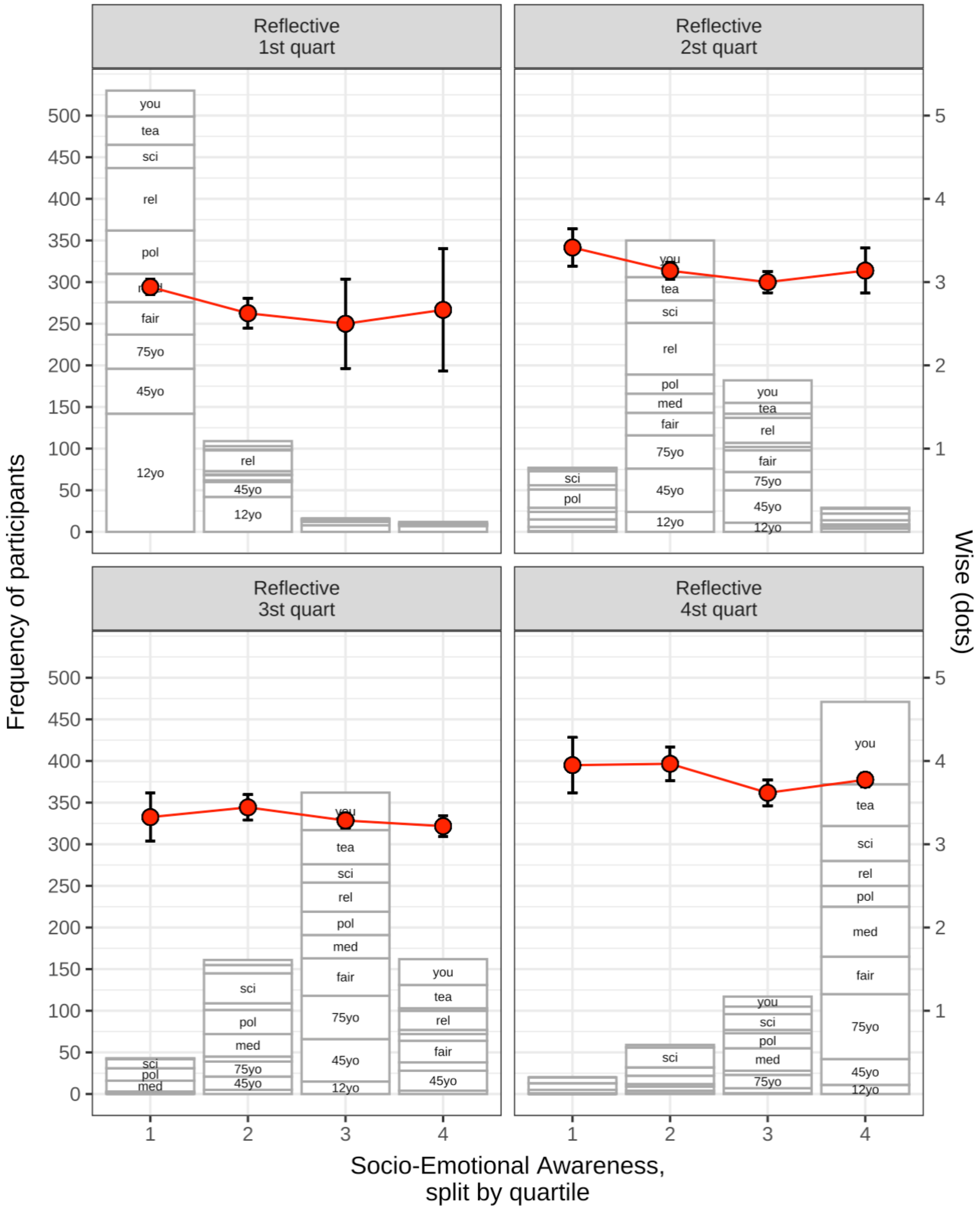

Figure S8. Representation of the interaction term as frequency of participants who gave different ratings of wisdom as well as the corresponding scores on Reflective Orientation and Socio-Emotional Awareness. Red line represents an average wisdom rating given the specific level of both dimensions. The quartiles are based on the between-level factor scores predicted by the pooled-sample isomorphic ML CFA.

Supplementary Note C – Target rankings

General ranking

Table S30 lists the effects of nine targets on the two latent dimensions of wisdom perception. Interestingly, a *politician* was perceived as average on Reflective Orientation and low on Socio-Emotional Awareness. Female targets compared to male ones were rated lower on Reflective Orientation and Socio-Emotional Awareness. Including gender as a covariate did not change the order of targets.

The overall ratings of the targets by wisdom, knowledgeability, and understanding were similar to each other (right-hand part of Table S30). "You" served as a reference category in the regressions, and effects of all the other targets were estimated relative to the answers about "you" and represent differences from answers about "you" (see Figure S9 for the comparison of "you" ratings). The *doctor*, *scientist*, and *75-year-old* were rated the wisest. Participants rated the *12-year-old* target the least wise, followed by the *religious* person. The self, *45-year-old* target and *politician* were rated essentially average (~3 points) on wisdom. *Politician* was rated as more knowledgeable, but less wise, and low on understanding, whereas the *fair person* was rated as very understanding, but less wise and knowledgeable. Controlling for gender did not change the order of the targets.

Table S30. Standardized regression coefficients of the two dimensions of wisdom perception and explicit ratings of wisdom, knowledgeability, and understanding on targets.

Independent variables	Reflective Orientation		Socio-Emotional Awareness		Wise		Knowledgeable		Understanding	
	β	*p*	*β*	*p*	*β*	*p*	*β*	*p*	*β*	*p*
You										
Religious	-.11	*	-.06	*	-.07	*	-.09	*	-.14	*
12-year-old	-.49	*	-.34	*	-.31	*	-.33	*	-.37	*
45-year-old	-.04	*	-.05	*	-.00	.719	-.03	*	-.06	*
75-year-old	.08	*	-.01	.461	.23	*	.17	*	.08	*
Politician	.10	*	-.19	*	.01	.277	.05	*	-.10	*
Doctor	.17	*	-.04	*	.25	*	.29	*	.11	*
Scientist	.14	*	-.09	*	.24	*	.31	*	.04	*
Fair	.04	*	.04	*	.08	*	.04	*	.06	*
Teacher	.09	*	.03	.001	.12	*	.15	*	.03	*
Target is female	-.02	.004	.01	.156	.01	.105	-.03	*	.02	.013
Target's gender is not specified	-.07	*	-.08	*	-.01	.073	-.04	*	.02	.010

Note. Pooled sample model controlling for the target's gender. Model fit: CFI = .916; TLI = .897; RMSEA = 0.027; $SRMR_{within}$ = .031; $SRMR_{between}$ = .063. *β* = unstandardized regression coefficient, *p* = *p*-value. * $p < .001$.

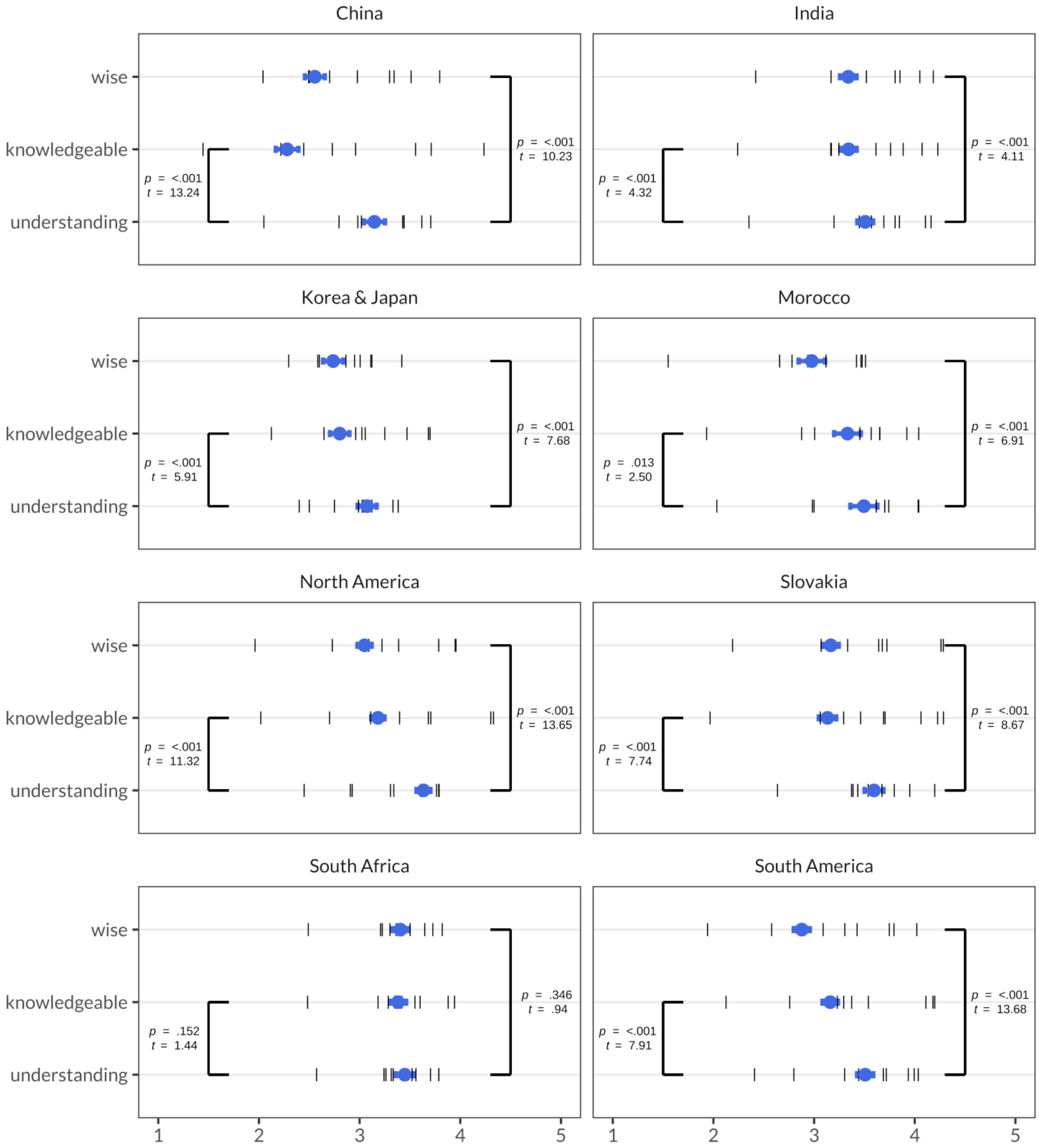

Figure S9. Average ratings of wisdom, knowledgeability, and understanding of self across cultural regions. Horizontal bar stands for 95% CI of the mean self-rating. Vertical bars represent the other targets.

Stability across cultural regions

In order to evaluate the stability of targets' ratings across cultural regions, we first estimated effects of each target on the two dimensions using a multiple group ML SEM assuming partial measurement invariance. Next, we extracted regression coefficients reflecting relative position of each target along the two dimensions for each group, and correlated these coefficients across groups. It resulted in twenty-eight correlation estimates (e.g., the target positions on Reflective Orientation in South and North America correlated r = .99 but .84 on Socio-Emotional Awareness). Figure S10 shows the distributions of intercorrelations between the targets' positions on the two dimensions. It is apparent that the cross-cultural stability of Reflective Orientation was substantially higher.

In a similar vein, average ratings of wisdom, knowledgeability, and understanding in each cultural region were highly stable. Across regions, an average intercorrelation of mean ratings of wisdom was r = .91, for knowledgeability r = .94, and it was a little lower for understanding, r = .80.

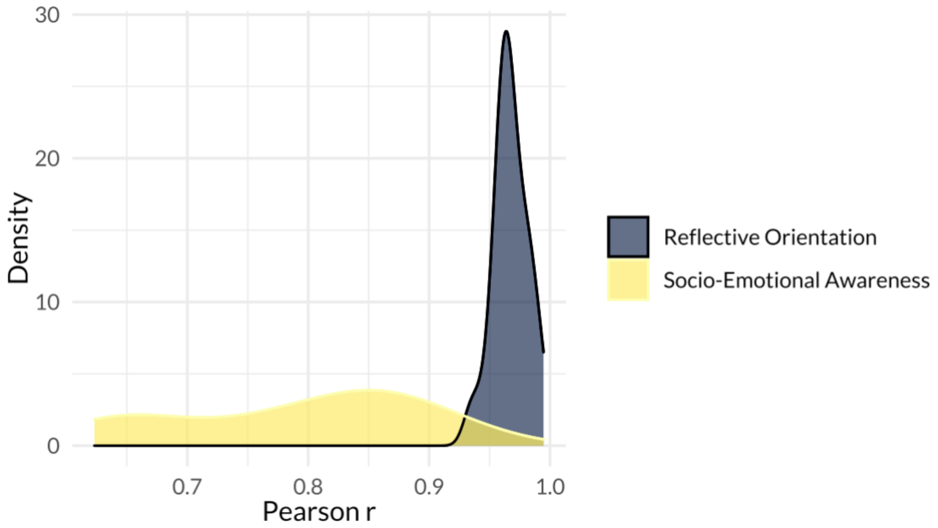

Figure S10. Distribution of intercorrelations between target's positions on the two latent dimensions across cultural regions

As an additional step, we obtained a model-based measure of the stability of the target's positions across cultural regions. We estimated a new parameter in the model. This parameter was a function of targets' effects on the two dimensions. First, we standardized the parameters by the variance of latent variables. Next, we computed a standard deviation for each target across regions to capture its variability, and then averaged these standard deviations across targets. Finally, we computed the difference of cross-regional variability between the two wisdom perception dimensions. The estimated parameters are listed in Table S31. An overall estimate of cross-regional variability of target's ratings (average *SD*) was .11, 95%CI [.07, .13] and .18, 95%CI [.16, .21] for Reflective Orientation and Socio-Emotional Awareness, with associated effect sizes of d = .13 and .19, respectively. These estimates suggest a moderate-level effect of cultural variability. Most of the variability of Reflective Orientations came from differences in the ratings of the *12-year-old, 45-year-old*, and *religious* person. The *politician*'s Socio-

Emotional Awareness varied the most across regions, followed by the Socio-Emotional Awareness of the 75-year-old, *scientist*, and *doctor*.

Notably, the difference in the variabilities of targets between Reflective Orientation and Socio-Emotional Awareness was .07 95%CI [.04, .10] with $d = .06$, which suggests that the ratings of targets by Reflective Orientation were more stable across cultures than the ratings by Socio-Emotional Awareness, although the size of the differences in stability was relatively small.

Table S31. Estimates of cross-regional variability in targets' ratings along the two perception dimensions.

Target	Reflective Orientation			Socio-Emotional Awareness			Difference		
	b	*p*	*d*	*b*	*p*	*d*	*b*	*p*	*d*
12-year-old	.22	*	.13	.18	*	.08	-.04	.191	-.02
45-year-old	.13	*	.08	.13	*	.07	-.00	.980	-.00
75-year-old	.08	*	.05	.19	*	.11	.11	*	.06
Doctor	.06	.011	.03	.20	*	.10	.14	*	.05
Fair	.12	*	.07	.12	*	.06	<|.01|	.961	-.00
Politician	.08	*	.05	.27	*	.14	.19	*	.07
Religious	.13	*	.08	.18	*	.09	.05	.071	.02
Scientist	.09	*	.05	.20	*	.10	.11	*	.06
Teacher	.05	.012	.03	.16	*	.09	.11	*	.05
Mean	.11	*	.13	.18	*	.19	.07	*	.06

Note. b = Estimate of cross-regional variability, p = p-value, d = effect size, *p-value <.001.

Supplementary Note D – Measurement invariance of the wisdom probes

To avoid ambiguities of the term "wisdom" and related difficulties of translation we used two extra probes of epistemic content referred to as "knowledgeable" and "understanding" in English. We also constructed a latent variable out of the three terms (wisdom, knowledge, understanding). The results listed in Table S32 show partial metric invariance across eight cultural regions. Factor loadings for all three items appeared to be similar across cultural regions – which points to similarity in meaning of these terms across eight cultural regions, with exception of the 'understanding' item at the within-person level: it showed stronger loadings in the global South sites (South America, South Africa, Morocco, India) and lower in the North (North America, Slovakia, Korea/Japan, and China), see Fig. S11. Accordingly, "understanding" has also slightly deviated in its associations with the two perception dimensions.

Table S32. Measurement invariance tests of the wisdom probes, Multiple group ML CFA model.

	*N*par	CFI	Δ	RMSEA	Δ	SRMR Between	SRMR Within	BIC
Configural	120	1		0		0	0	193039.1
Full metric invariance	92	0.958	.042	0.060	.060	.082	.028	193419.8
Partial metric invariance (all loadings but understanding at the within level are invariant)	99	0.993	.007	0.027	.027	.072	.008	193007.8
Full scalar invariance	78	0.922	.070	0.066	.006	.105	.028	193684.7
Partial scalar invariance (all loadings but understanding at the within level and the intercept of knowledgeability are invariant)	92	0.977	.016	0.044	.017	.085	.008	193144.9

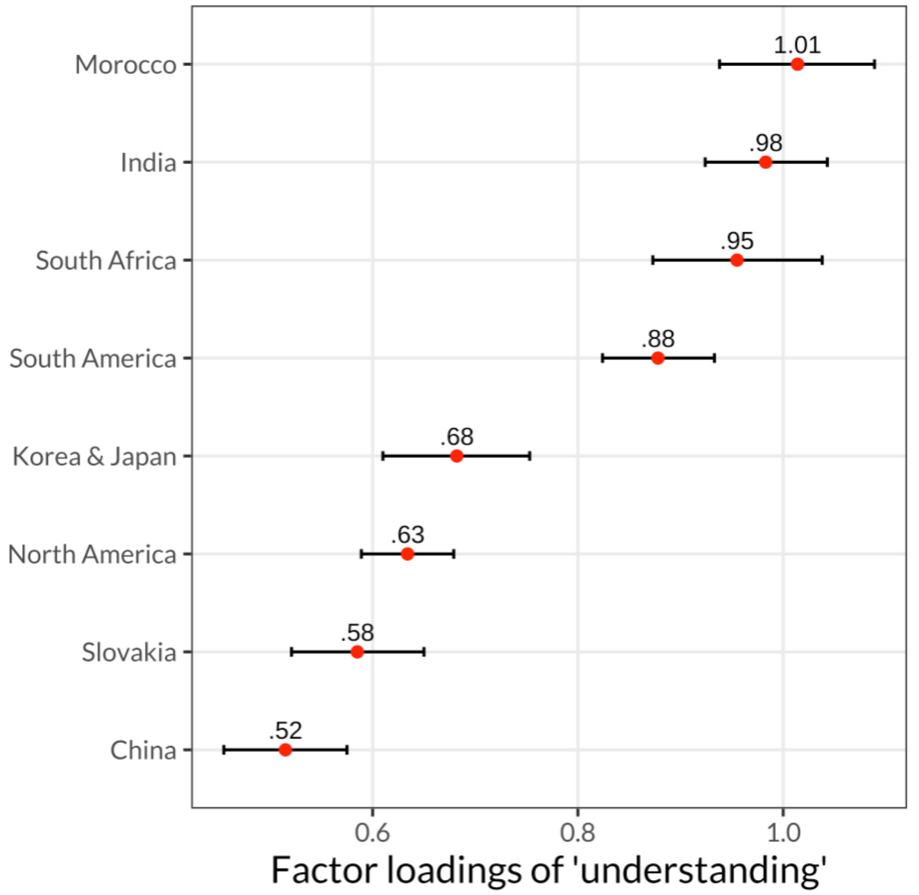

Figure S11. Factor loadings of ‘understanding’ estimated at the within level by the partial metric multi-group ML CFA across eight cultural groups where items ‘wise’ and ‘knowledgeable’ are invariant.

Supplementary References

1. Weststrate, N. M., Ferrari, M. & Ardelt, M. The many faces of wisdom: An investigation of cultural-historical wisdom exemplars reveals practical, philosophical, and benevolent prototypes. *Personality and Social Psychology Bulletin* **42**, 662–676 (2016).
2. Depaoli, S. & van de Schoot, R. Improving transparency and replication in Bayesian statistics: The WAMBS-Checklist. *Psychological Methods* **22**, 240–261 (2017).
3. Gelman, A., Carlin, J. B., Stern, H. S. & Rubin, D. B. *Bayesian Data Analysis*. (Chapman and Hall/CRC, 1995).
4. R Core Team. *R: A Language and Environment for Statistical Computing*. (R Foundation for Statistical Computing, Vienna, Austria, 2023).
5. Wilke, C. O. *Cowplot: Streamlined Plot Theme and Plot Annotations for 'Ggplot2'*. (2020).
6. Wickham, H., François, R., Henry, L., Müller, K. & Vaughan, D. *Dplyr: A Grammar of Data Manipulation*. (2023).
7. Pedersen, T. L. *Ggraph: An Implementation of Grammar of Graphics for Graphs and Networks*. (2022).
8. Slowikowski, K. *Ggrepel: Automatically Position Non-Overlapping Text Labels with 'Ggplot2'*. (2024).
9. Wilke, C. O. & Wiernik, B. M. *Ggtext: Improved Text Rendering Support for 'Ggplot2'*. (2022).
10. Tay, J. K., Narasimhan, B. & Hastie, T. Elastic Net Regularization Paths for All Generalized Linear Models. *Journal of Statistical Software* **106**, 1–31 (2023).
11. Auguie, B. *gridExtra: Miscellaneous Functions for 'Grid' Graphics*. (2017).
12. Arnold, T. B. *Hdlm: Fitting High Dimensional Linear Models*. (2016).
13. Zhu, H. *kableExtra: Construct Complex Table with 'kable' and Pipe Syntax*. (2021).
14. Xie, Y. *Knitr: A General-Purpose Package for Dynamic Report Generation in R*. (2023).
15. Rudnev, M. LittleHelpers: Helpers for comparative studies. (2024).
16. Hallquist, M. N. & Wiley, J. F. MplusAutomation: An R Package for Facilitating Large-Scale Latent Variable Analyses in Mplus. *Structural Equation Modeling* 621–638 (2018) doi:10.1080/10705511.2017.1402334.
17. William Revelle. *Psych: Procedures for Psychological, Psychometric, and Personality Research*. (Northwestern University, Evanston, Illinois, 2023).
18. Epskamp, S., Cramer, A. O. J., Waldorp, L. J., Schmittmann, V. D. & Borsboom, D. qgraph: Network Visualizations of Relationships in Psychometric Data. *Journal of Statistical Software* **48**, 1–18 (2012).
19. Wickham, H. Reshaping Data with the reshape Package. *Journal of Statistical Software* **21**, 1–20 (2007).
20. Qiu, Y. *Showtext: Using Fonts More Easily in R Graphs*. (2023).
21. Pedersen, T. L. *Tidygraph: A Tidy API for Graph Manipulation*. (2023).
22. Wickham, H., Vaughan, D. & Girlich, M. *Tidyr: Tidy Messy Data*. (2024).
23. Henry, L. & Wickham, H. *Tidyselect: Select from a Set of Strings*. (2022).
24. Brown, T. A. *Confirmatory Factor Analysis for Applied Research*. (Guilford publications, 2015).
25. Weijters, B., Geuens, M. & Schillewaert, N. The individual consistency of acquiescence and extreme response style in self-report questionnaires. *Applied psychological measurement* **34**, 105–121 (2010).
