# Supplementary material for: Dimensions of wisdom perception across twelve countries on five continents
Source: Nat Commun. 2024 Aug 14;15:6375. doi: 10.1038/s41467-024-50294-0 (PMC11324649; doi:10.1038/s41467-024-50294-0)
Supplement: Supplementary file 2 — Reporting Summary [file 41467_2024_50294_MOESM2_ESM.pdf]

nature portfolio

Corresponding author(s): Maksim Rudnev

Last updated by author(s): May 21, 2024

# Reporting Summary

Nature Portfolio wishes to improve the reproducibility of the work that we publish. This form provides structure for consistency and transparency in reporting. For further information on Nature Portfolio policies, see our Editorial Policies and the Editorial Policy Checklist.

## Statistics

For all statistical analyses, confirm that the following items are present in the figure legend, table legend, main text, or Methods section.

| n/a | Confirmed | |
|---|---|---|
| ☐ | ☒ | The exact sample size (*n*) for each experimental group/condition, given as a discrete number and unit of measurement |
| ☐ | ☒ | A statement on whether measurements were taken from distinct samples or whether the same sample was measured repeatedly |
| ☐ | ☒ | The statistical test(s) used AND whether they are one- or two-sided<br>*Only common tests should be described solely by name; describe more complex techniques in the Methods section.* |
| ☐ | ☒ | A description of all covariates tested |
| ☐ | ☒ | A description of any assumptions or corrections, such as tests of normality and adjustment for multiple comparisons |
| ☐ | ☒ | A full description of the statistical parameters including central tendency (e.g. means) or other basic estimates (e.g. regression coefficient) AND variation (e.g. standard deviation) or associated estimates of uncertainty (e.g. confidence intervals) |
| ☐ | ☒ | For null hypothesis testing, the test statistic (e.g. *F*, *t*, *r*) with confidence intervals, effect sizes, degrees of freedom and *P* value noted<br>*Give P values as exact values whenever suitable.* |
| ☐ | ☒ | For Bayesian analysis, information on the choice of priors and Markov chain Monte Carlo settings |
| ☐ | ☒ | For hierarchical and complex designs, identification of the appropriate level for tests and full reporting of outcomes |
| ☐ | ☒ | Estimates of effect sizes (e.g. Cohen's *d*, Pearson's *r*), indicating how they were calculated |

*Our web collection on statistics for biologists contains articles on many of the points above.*

## Software and code

Policy information about availability of computer code

| | |
|---|---|
| Data collection | some samples were collected using Qualtrics online platform |
| Data analysis | Most models were calculated in Mplus 8.8 software (Muthén & Muthén, 2022). Summaries and extra analyses were run within R 4.1 (R Core team, 2023) Code is available https://osf.io/m4dxv/ |

For manuscripts utilizing custom algorithms or software that are central to the research but not yet described in published literature, software must be made available to editors and reviewers. We strongly encourage code deposition in a community repository (e.g. GitHub). See the Nature Portfolio guidelines for submitting code & software for further information.

## Data

Policy information about availability of data

All manuscripts must include a data availability statement. This statement should provide the following information, where applicable:

- Accession codes, unique identifiers, or web links for publicly available datasets
- A description of any restrictions on data availability
- For clinical datasets or third party data, please ensure that the statement adheres to our policy

The data is shared in an OSF diretory https://osf.io/m4dxv

## Research involving human participants, their data, or biological material

Policy information about studies with human participants or human data. See also policy information about sex, gender (identity/presentation), and sexual orientation and race, ethnicity and racism.

| | |
|---|---|
| Reporting on sex and gender | Gender of participants was indicated by participants' self-identification. Findings apply to gender only. |
| Reporting on race, ethnicity, or other socially relevant groupings | The study focused on perceptions of people without referring to their race or ethnicity. Participants' race and ethnicity were not involved in the study either. |
| Population characteristics | See above |
| Recruitment | Participant recruitment differed across countries, see https://osf.io/m4dxv Because we relied on convenience sampling, participants were not representative of the populations in their respective cultural regions, and the self-selection bias could be present. |
| Ethics oversight | This study has been reviewed and received ethics clearance through the University of Waterloo Research Ethics Committee (ORE #42159), and by the corresponding ethics body in each of the ten other countries involved in the study, see https://osf.io/m4dxv |

Note that full information on the approval of the study protocol must also be provided in the manuscript.

# Field-specific reporting

Please select the one below that is the best fit for your research. If you are not sure, read the appropriate sections before making your selection.

☐ Life sciences ☒ Behavioural & social sciences ☐ Ecological, evolutionary & environmental sciences

For a reference copy of the document with all sections, see nature.com/documents/nr-reporting-summary-flat.pdf

# Behavioural & social sciences study design

All studies must disclose on these points even when the disclosure is negative.

| | |
|---|---|
| Study description | Quantitative cross-sectional cross-cultural study |
| Research sample | Convenience samples across 16 sites in 11 countries via the Qualtrics online platform or via paper-and-pencil (in Slovakia and Morocco; see Table 1). Samples from Canada, Ecuador, Peru, and the US consisted of university students, the other samples came from a broader population. |
| Sampling strategy | Convenience sampling |
| Data collection | Data was collected between 2019 and 2021 from convenience samples across 16 sites in 11 countries via the Qualtrics online platform or via paper-and-pencil (in Slovakia and Morocco; see Table 1). Samples from Canada, Ecuador, Peru, and the US consisted of university students, the other samples came from a broader population. |
| Timing | between 2019 and 2021 |
| Data exclusions | No data was excluded |
| Non-participation | Such information was not collected |
| Randomization | Conditions were randomized. Participants were randomly assigned to only one reference target from the list of ten (between-subject element), to which they compared all other targets (within-subject element; presented in a pseudo-random order). |

# Reporting for specific materials, systems and methods

We require information from authors about some types of materials, experimental systems and methods used in many studies. Here, indicate whether each material, system or method listed is relevant to your study. If you are not sure if a list item applies to your research, read the appropriate section before selecting a response.

## Materials & experimental systems

| n/a | Involved in the study |
|---|---|
| ☒ | ☐ Antibodies |
| ☒ | ☐ Eukaryotic cell lines |
| ☒ | ☐ Palaeontology and archaeology |
| ☒ | ☐ Animals and other organisms |
| ☒ | ☐ Clinical data |
| ☒ | ☐ Dual use research of concern |
| ☒ | ☐ Plants |

## Methods

| n/a | Involved in the study |
|---|---|
| ☒ | ☐ ChIP-seq |
| ☒ | ☐ Flow cytometry |
| ☒ | ☐ MRI-based neuroimaging |
